# Hippocampal structure, patterns of the calcium-binding proteins and neuron numbers in small echolocating bats

**DOI:** 10.3389/fnana.2025.1641787

**Published:** 2025-08-13

**Authors:** Jovana Maliković, Katja Schönbächler, Ana Luiza F. Destro, David P. Wolfer, Irmgard Amrein

**Affiliations:** ^1^Division of Functional Neuroanatomy, Institute of Anatomy, University of Zürich, Zürich, Switzerland; ^2^Bat Conservation Switzerland, Zürich, Switzerland; ^3^Department of Animal Biology, Federal University of Viçosa, Viçosa, Brazil; ^4^D-HEST, Institute of Human Movement Sciences and Sport, ETH Zürich, Zürich, Switzerland

**Keywords:** Chiroptera, calcium-binding protein, stereology, comparative, hippocampus, mossy fibers, ecology, diet

## Abstract

Even though bats are the second most speciose group of mammals, neuroanatomical studies of their hippocampus are rare, particularly of small echolocating bats. Here, we provide a qualitative and quantitative neuroanatomical analysis of the hippocampus of small echolocating bats (Phyllostomidae and Vespertilionidae). Calcium-binding proteins revealed species- and family-specific patterns for calbindin and calretinin. Interneuron staining for both proteins was very rare in phyllostomids, while calretinin marked subpopulations of CA3 pyramidal neurons in both families. Parvalbumin expression was consistent across bats and similar to other species. A unique calretinin-positive calbindin-negative zone was observed at the superficial boundary of the CA3 pyramidal cell layer in phyllostomid bats. This zone defined a gap between pyramidal cells and the zinc-positive mossy fibers. We hypothesize that this gap might either stem from calretinin-positive afferents displacing the zinc-positive mossy fiber boutons, or from a complete segregation of neurochemically distinct mossy boutons. Furthermore, we observed a distinct dorsoventral shift in the length of the upper and lower blade of the granule cell layer in all species. In terms of hippocampal neuron numbers, bats were characterized by a rather small granule cell and subicular neuron population, but a well-developed CA3. In a correspondence analysis, preferred diet segregated phyllostomids into a hilus-dominant omnivorous and frugivorous species group, and a subiculum-dominant group containing vampire bats and nectivorous species. Although the two families overlapped considerably, the cellular composition of the phyllostomid hippocampus can be described as output dominant, while in vespertilionids neuron populations on the hippocampal input side are more dominant. Neuroanatomical and ecological variability and unique traits within echolocating bats as shown here can provide a rich source for investigating structure-function relationships.

## 1 Introduction

Describing common anatomical hippocampal characteristics across all bats poses a considerable challenge due to the significant differences observed between large fruit bats (Yinpterochiroptera) and small echolocating bats (Yangochiroptera) (Baron et al., 1996c). The hippocampus exhibits a relatively uniform cytoarchitecture within the group of large fruit bats, while small echolocating bats show hippocampal variations, not only in comparison to large fruit bats but, in particular in the CA1 region, also among themselves (Stephan et al., 1987; Baron et al., 1996a,b). While large fruit bats have garnered attention in anatomical (Buhl and Dann, 1991; Gatome et al., 2010; Eilam-Altstädter et al., 2021; Jacobsen et al., 2023) and functional studies (Sarel et al., 2017; Eliav et al., 2021; Ray et al., 2025), there is no recent anatomical study on the hippocampus of small echolocating bats. This is despite early evidence of a specialization in the form of a radially expanded and highly differentiated CA1 pyramidal cell layer (Stephan et al., 1987) that is similar and common in primates, including humans (Rosene and van Hoesen, 1987; Slomianka et al., 2011). Stephan (1975) proposed that the gradual dispersal of a compact CA1 pyramidal cell layer, from insectivores to humans, may represent stages of evolution. However, this phylogenetic interpretation of CA1 cytoarchitecture does not consider that the dispersal of CA1 pyramidal cells into the stratum oriens is present in a wide range of taxonomically diverse species (Jacobs et al., 1979; Hof et al., 1996; Slomianka et al., 2011; Maliković et al., 2023).

The ecology of small echolocating bats is as varied as the structure of their hippocampi, providing opportunities to look for relations between structure, function and life style. Many of the ecological differences relate to the use of (aerial) space and foraging strategies (Norberg and Rayner, 1987; Denzinger and Schnitzler, 2013): some are aerial hunters, capturing flying prey in open space or at the edges of vegetation, while others are narrow space gleaners, collecting their preferred food from the ground, foliage, or water surfaces. Some species rely on a vegetarian diet, consuming fruits, nectar, and pollen. Few species are exclusive blood feeders, while others prey on invertebrates, vertebrates, or adopt an omnivorous diet. Even though the proportions of the major brain divisions change in clade-dependent patterns, ecological niches associate with similar proportions of brain components across diverse mammalian species groups (de Winter and Oxnard, 2001). Relations between hippocampal volume and lifestyle/ecology in echolocating bats have been tested, (Baron et al., 1996b; Hutcheon et al., 2002; Safi and Dechmann, 2005; Ratcliffe et al., 2006) but findings were not conclusive. Beyond changes in the proportions of major brain divisions, there is little evidence on how the internal organization of these divisions, including the hippocampus, may change to accommodate different functional demands. The contribution of each of the five principal hippocampal neuron population to the main functions of the hippocampus, that is encoding of space, time and memory, has been well-described (Eichenbaum, 2017; Rebola et al., 2017; Matsumoto et al., 2019; Borzello et al., 2023), and there is substantial evidence for such functions in bats too. Electrophysiological recordings from hippocampal CA1 neurons detailed the complex sociospatial coding in freely moving Egyptian fruit bats (*Rousettus aegyptiacus*) (Forli and Yartsev, 2023; Ray et al., 2025), and showed remapping in CA1 and subiculum in response to switching sensory modalities (Geva-Sagiv et al., 2016). Similar findings were presented for an echolocating bat, the big brown bat (*Eptesicus fuscus*), where hippocampal CA1 pyramidal neurons showed spatial (Ulanovsky and Moss, 2007) and acoustic sensitivity (Yu and Moss, 2022). The often very small size of echolocating bats makes such studies challenging, but macromorphological brain characteristics (van Tussenbroek et al., 2023), combined with knowledge of the morphological and quantitative makeup of the hippocampus as provided here, can identify promising species for further studies.

In this comparative study, we present details of the hippocampus in vespertilionids (nine species) and phyllostomids (six species). Analyzed species are *Plecotus auritus* (brown long-eared bat), *Pipistrellus pipistrellus* (common pipistrelle), *Pipistrellus nathusii* (Nathusius’ pipistrelle), *Pipistrellus kuhlii* (Kuhl’s pipistrelle), *Pipistrellus pygmaeus* (soprano pipistrelle), *Vespertilio murinus* (parti-colored bat), *Myotis daubentonii* (Daubenton’s bat), *Myotis mystacinus* (whiskered bat), *Myotis nigricans* (black myotis), *Desmodus rotundus* (common vampire bat), *Diphylla ecaudata* (hairy-legged vampire bat), *Anoura caudifer* (tailed tailless bat), *Sturnira lilium* (little yellow-shouldered bat), *Carollia perspicillata* (Seba’s short-tailed bat) and *Phyllostomus discolor* (pale spear-nosed bat). We illustrate the basic cytoarchitectural characteristics seen in Nissl stains and describe the distributions of the calcium-binding proteins (CaBPs) calbindin, calretinin, and parvalbumin. CaBPs are used as markers for subpopulations of both cortical and hippocampal interneurons and principal neurons (DeFelipe, 1993; Freund and Buzsáki, 1996; Slomianka et al., 2013; Medalla et al., 2023). The comparative assessment of CABPs allows the definition of subpopulations and provides cues if CaBP expression is likely to serve basic hippocampal function across clades or if it is more likely to serve clade- or species-specific demands on hippocampal information processing. These CaBPs are also helpful in defining the boundaries of hippocampal subfields (Rami et al., 1987; Ashwell et al., 2008; Maliković et al., 2024), which facilitates robust neuron number estimates. We present neuron numbers of the five principal hippocampal neuron populations: granule cells (GC), hilar neurons (HIL), CA3 and CA1 pyramidal neurons (CA3 and CA1 respectively), and subicular neurons (SUB), in this sample of small echolocating bats. Neuron numbers are visualized and discussed in relation to phylogenetic clustering, foraging habitat and diet.

## 2 Materials and methods

### 2.1 Animals and tissue preparation

Brains were collected from different sources (Table 1). Samples were obtained during field work for other experimental purposes, collected from animals euthanized due to the severe injury, or were available from previous work (Amrein et al., 2007).

**TABLE 1:** Species list.

Latin name	Common name	Family	Brain weight (g)	Body weight (g)	Diet	Foraging habitat	Permit/ source
*Myotis daubentonii*	Daubenton’s bat	Vespertilionidae	0.19	7.0	Insectivorous	Edge space	1
*Myotis mystacinus*	Whiskered bat	Vespertilionidae	0.12	3.9	Insectivorous	Edge space	1
*Myotis nigricans*	Black myotis	Vespertilionidae	0.12	2.9	Insectivorous	Edge space	2
*Pipistrellus kuhlii*	Kuhl’s pipistrelle	Vespertilionidae	0.11	5.5	Insectivorous	Edge space	1
*Pipistrellus nathusii*	Nathusius’ pipistrelle	Vespertilionidae	0.14	6.8	Insectivorous	Edge space	1
*Pipistrellus pipistrellus*	Common pipistrelle	Vespertilionidae	0.10	4.4	Insectivorous	Edge space	1
*Pipistrellus pygmaeus*	Soprano pipistrelle	Vespertilionidae	0.11	5.1	Insectivorous	Edge space	1
*Plecotus auritus*	Brown long-eared bat	Vespertilionidae	0.22	10.2	Insectivorous	Edge space	1
*Vespertilio murinus*	Parti-colored bat	Vespertilionidae	0.16	10.0	Insectivorous	Open space	1
*Anoura caudifer*	Tailed tailless bat	Phyllostomidae	0.13	12.5	Nectivorous	Narrow space	2
*Carollia perspicillata*	Seba’s short-tailed bat	Phyllostomidae	0.59	25.0	Frugivorous	Narrow space	4
*Desmodus rotundus*	Common vampire bat	Phyllostomidae	0.89	36.9	Hematophagous	Narrow space	2
*Diphylla ecaudata*	Hairy-legged vampire bat	Phyllostomidae	0.62	30.1	Hematophagous	Narrow space	2
*Phyllostomus discolor*	Pale-spear nosed bat	Phyllostomidae	1.03	38.0	Omnivores	Narrow space	3
*Sturnira lilium*	Little yellow-shouldered bat	Phyllostomidae	0.35	24.7	Nectivorous	Narrow space	2

Brain and body weights are given as species mean. Permit/source: (1) Permit # WHgH2019060, issued by the Canton Zurich veterinary office, Switzerland/Bat Conservation Switzerland, Zürich, Switzerland. (2) Permit # 77787-4 issued by Ministry of environmental protection (MMA), Brazil/Department of Animal Biology, Federal University of Viçosa, Minas Gerais, Brazil (non-CITES). (3) Permit # 55.2-1-54-2531-128-08 and 55.2-2532.Vet_02-16-37 issued by the Regierung von Oberbayern, Germany/TUM School of Life Sciences, Technical University of Munich, Freising, Germany (non-CITES). (4) Amrein et al. (2007).

Brains were removed from the cranial cavity 5 min–2 h postmortem and immersion-fixed in 4% phosphate-buffered paraformaldehyde containing 15% picric acid. For immunohistochemistry, one hemisphere was cryoprotected in a 30% sucrose solution, frozen and cut horizontally at a thickness of 40 μm using a sliding microtome (Microm, HM325). Serial sections were collected and preserved in cryoprotectant at −20°C. For the quantitative assessment of hippocampal neuron numbers, one hemisphere was embedded in 2-hydroxyethyl-methacrylate (2-HEMA; Technovit 7100, Heraeus Kulzer GmbH, Wehrheim/Ts, Germany) following the manufacturer’s instructions. Tissue sections were cut horizontally at 20 μm, mounted, and dried at 60°C for 1 h. Giemsa, a modified Nissl stain, was performed according to the protocol of Iñiguez et al. (1985).

### 2.2 Immunohistochemistry for calbindin, calretinin and parvalbumin

For the qualitative assessment of hippocampal features, immunohistochemical staining were performed in all species except in *Pipistrellus pygmaeus*, *Plecotus auritus* and *Sturnira lilium* where only HEMA-embedded material was available. We used free-floating sections of series that spanned the entire hippocampus. Negative controls for non-specific binding were routinely performed by omitting the primary antibody. Stained neuronal elements resembled those observed in mouse sections processed in the same batches and reported for other species. However, without knockout controls, all immunoreactivity that we report here should be read as calbindin-like, calretinin-like or parvalbumin-like.

Epitopes were retrieved with 0.5% sodium borohydride in phosphate-buffered saline (PBS) for 30 min. Endogenous peroxidase was blocked with 0.6% hydrogen peroxide in Tris-Triton-buffer (1:10 of Tris base in dH_2_0 + 0.05% of Triton, ph 7.4) for 15 min. Subsequently, sections were incubated for 1 h in 2% normal serum (calbindin and calretinin: goat, parvalbumin: horse) with 0.2% Triton in Tris-Triton buffer. Next, sections were incubated overnight at room temperature using rabbit anti-calretinin (Swant, CR7697, Lot 1893-0114, dilution 1:500), rabbit anti-calbindin (Swant, CB-38a, Lot 9.03, dilution 1:500) and mouse anti-parvalbumin (Sigma Aldrich, P3088, Lot 104780. Dilution 1:1000). Afterward, sections were washed in Tris-buffered saline (TBS) and incubated with goat anti-rabbit (1:300, Vector labs, BA-1000, Lot X11041) or horse anti-mouse (1:300, Vector labs, BA-2000, Lot ZF0521) for 40 min at room temperature. After this step, sections were incubated for 20 min in avidin-biotin complex (Vector Labs, Lot ZJ0909) in TBS. Sections were 3,3′-Diaminobenzidine stained, mounted, dehydrated and cover-slipped using Eukitt.

### 2.3 Timm staining in phyllostomid bats

Timm stained sections of Seba’s short-tailed bat (*Carollia perspicillata*), pale spear-nosed bat (*Phyllostomus discolor*) and Pallas’s long-tongued bat (*Glossophaga soricina*) were available from a previous study (Amrein et al., 2007). From *Glossophaga soricina*, only the Timm stain was available for this study. In short, anesthetized animals were perfused transcardiacally in a series of PBS, 0.6% sodium sulfide solution and 4% paraformaldehyde. Horizontal cryostat sections (40 μm) were mounted and developed at 37°C in darkness for ∼60 min in a mixture of gum Arabic, hydroquinone and citric acid containing silver nitrate according to the protocol of Danscher and Zimmer (1978). Afterward, slides were rinsed in 1% sodium thiosulfate for 1 min, counterstained with neutral red, dehydrated and cover-slipped.

### 2.4 Definitions of hippocampal neuron populations

The naming and boundaries for hippocampal neuron populations followed conventions detailed in Maliković et al. (2023). In this study, the boundary between the CA3 and CA1 subregions was identified by the termination of the mossy fiber zone visible in Giemsa-stained sections. Definitions of neuron population boundaries were cross-checked using the stains for calcium-binding proteins, which often mark interregional boundaries.

### 2.5 Hippocampal neuron number estimation

Neuron number estimation was performed in the HEMA embedded, Nissl-stained sections of all 15 species (Table 1) using StereoInvestigator 10 Software (MBF Bioscience, Williston, VT, United States). In the Nissl stain, cytoplasm of neurons stains blue, nucleoli dark blue while the nucleus is largely unstained, differentiating neurons from glia as the cytoplasm of glia cells is not stained and nuclei stain light to dark blue without a distinct nucleolus (Fitting et al., 2010; García-Cabezas et al., 2016). Neuron counts were obtained using the optical fractionator method (West et al., 1991; Slomianka, 2021), with 10 μm high disector and a 2 μm top guard zone. Section thickness was measured at every fifth sampling site. Neuron counting was conducted under a × 63 oil immersion lens (NA 1.4). All sampling parameters and neuron counts are listed in the Supplementary Table 1.

Estimated total neurons were calculated based on number-weighted section thickness (Dorph-Petersen et al., 2001) and the precision of number estimates was assessed by the coefficient of error CE (Gundersen et al., 1999) with a conservative smoothness factor of m = 0.

### 2.6 Ecological characterization of the sampled bat species

Species classification for foraging habitat and dietary preferences (Table 1) were based on previous studies (Fleming, 1982; Norberg and Rayner, 1987; Ratcliffe, 2009; Denzinger and Schnitzler, 2013; Graf and Fischer, 2021).

### 2.7 Data analysis

Quantitative relations of each hippocampal neuron population (in percent) were visualized on the extracted phylogenetic tree (Álvarez-Carretero et al., 2022) using the R packages *ape* (Paradis and Schliep, 2019), *phytools* (Revell, 2024), *ggtree* (Yu et al., 2018) and *ggplot2* (Wickham, 2016). Hippocampal neuron numbers of each specimen were log_10_-transformed and z-scored (mean of 0 and standard deviation of 1 across all neuron populations), resulting in values representing the relative numeric contribution of each neuron population within the hippocampal circuitry. Z-scored neuron numbers were then visualized with a correspondence analysis as described before (Slomianka et al., 2013; Maliković et al., 2023) using the R package *made4* (Culhane et al., 2005), including factors such as species, family, diet preference and foraging habitat.

### 2.8 Imaging

Unless stated otherwise in the figure legends, images represent the intermediate (mid-dorsoventral) hippocampus and nearby structures in horizontal sections. Images were captured using a Zeiss Axio Imager.M2 microscope in the slide scanning mode of Stereo Investigator version 10 (MBF Bioscience, Williston, VT, RRID), using a × 20 objective.

## 3 Results

### 3.1 Hippocampal cytoarchitecture in vespertilionid and phyllostomid bats

**FIGURE 1:**
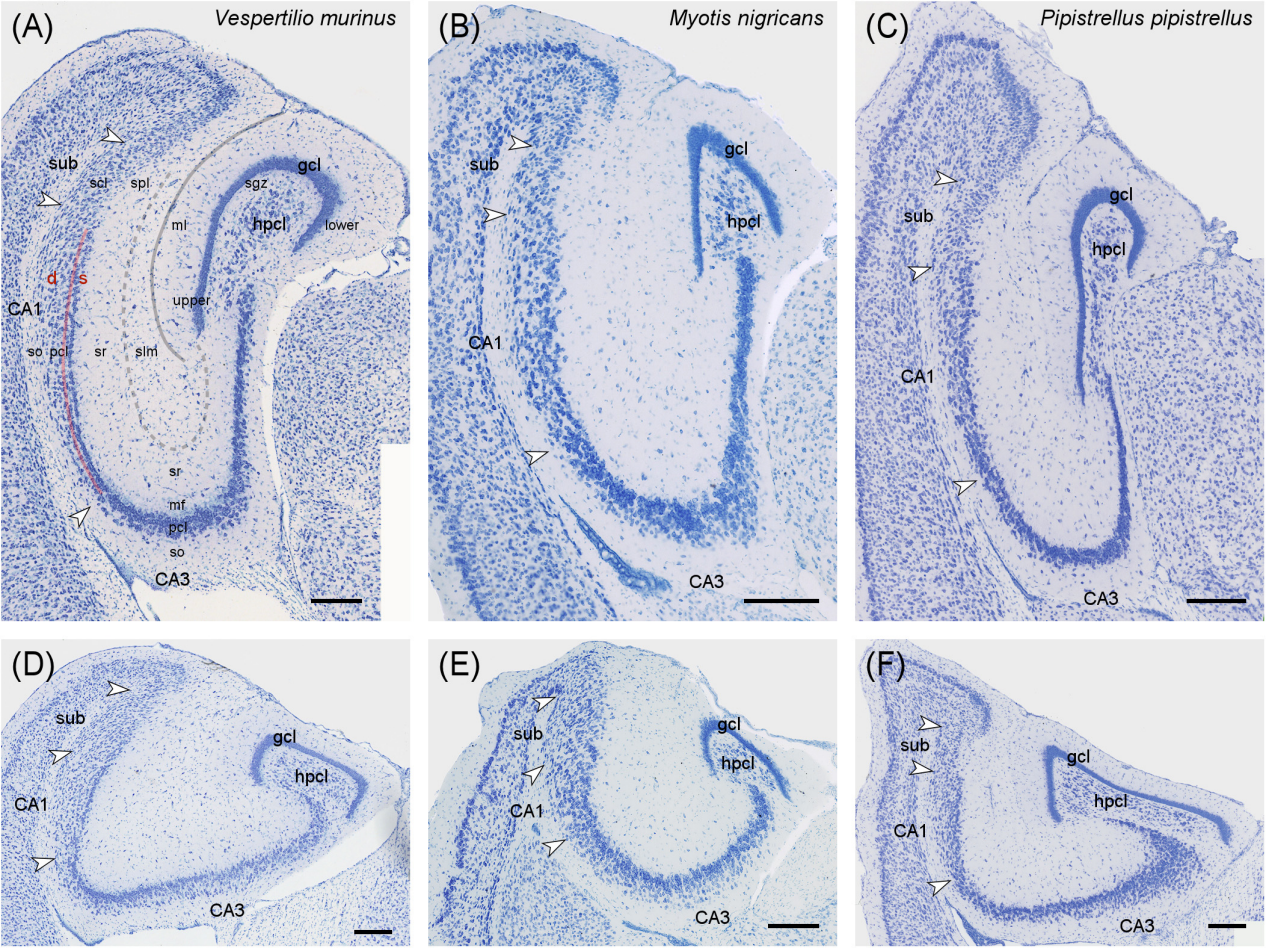
Hippocampal cytoarchitecture of vespertilionid bats. Three species covering the cytoarchitectural variations seen in vespertilionids are presented at the hippocampal intermediate **(A–C)** and ventral **(D–F)** level in Nissl-stained horizontal sections. **(A)** In *Vespertilio murinus* the characteristics of the vespertilionid hippocampus can be exemplified, with the granular cell layer (gcl) forming a dense band with an elongated upper blade dorsally and an elongated lower blade in the ventral hippocampus. The hilar polymorphic layer (hpcl) is separated by a cell-poor subgranular zone (sgz) from the gcl. The CA3 pyramidal cell layer is more compact dorsally than ventrally. The CA1 pyramidal cell layer shows the bat-typical loose arrangement, with neurons dispersing into stratum oriens (so). Superficial (s) CA1 neurons are more densely packed than deep (d) neurons. Transition to the subiculum (sub) is defined by neuron size, the presence of series of elongated, horizontally oriented neurons at the alveus border and the even dispersal of neurons. **(B)** In *Myotis nigricans*, blade length differences are small at the intermediate level and modest in the ventral region. **(C)** In *Pipistrellus pipistrellus*, blade differences are quite remarkable. Scale bar: 200 μm. White arrows mark neuron population boundaries between CA3, CA1 and subiculum. gcl, granule cell layer; hpcl, hilar polymorphic layer; CA3, area CA3; CA1, area CA1; sub, subiculum; upper blade of gcl; lower blade of gcl; s, superficial; d, deep; ml, molecular layer of the dentate; sgz, subgranular zone; so, stratum oriens; pcl, pyramidal cell layer; mf: mossy fiber zone; sr, stratum radiatum; slm: stratum lacunosum-moleculare; scl, subicular cell layer; spl, subicular plexiform layer; solid gray line, hippocampal fissure; dashed gray line, boundary between sr and slm; red line, separation between superficial and deep CA1 pyramidal neurons.

**FIGURE 2:**
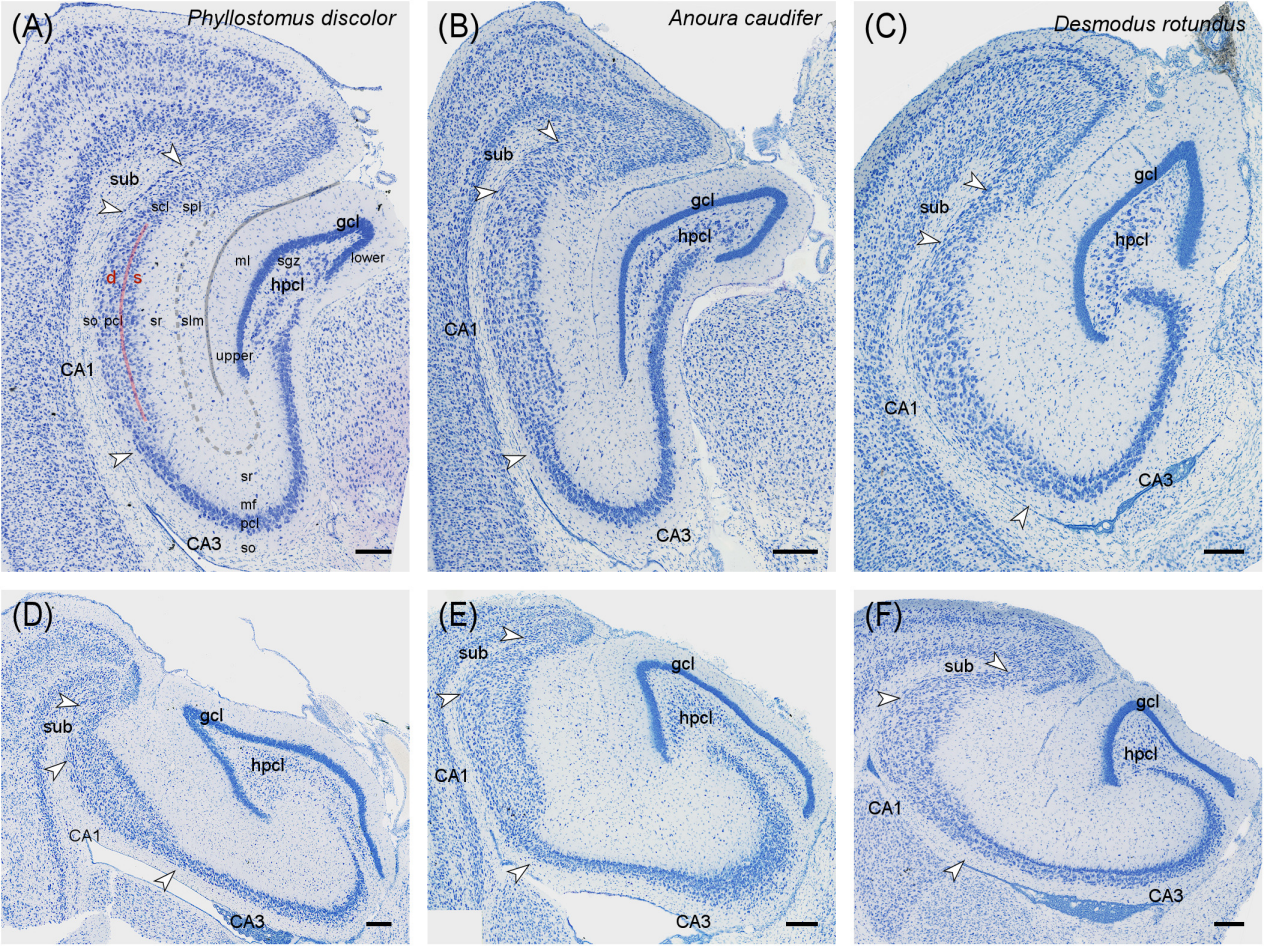
Hippocampal cytoarchitecture of phyllostomid bats. Three species covering the cytoarchitectural variations seen in phyllostomid bats are presented at the hippocampal intermediate **(A–C)** and ventral **(D–F)** level in Nissl-stained horizontal sections. **(A,D)** *Phyllostomus discolor* is the species with the characteristic appearance of this group. The cell-poor subgranular zone is wider than in vespertilionids and neurons of the hpcl are more densely arranged. The transition between CA3 and CA1 is rather sharp. **(B,E)** In *Anoura caudifer* the expansion of CA1 is most pronounced, with superficial neurons dispersing into stratum radiatum toward the hippocampal fissure. **(C,D)** Condensation of superficial CA1 pyramidal neurons was least prominent in *Desmodus rotundus*. Scale bar: 200 μm. White arrows mark neuron population boundaries between CA3, CA1 and subiculum. gcl, granule cell layer; hpcl, hilar polymorphic layer; CA3, area CA3; CA1, area CA1; sub, subiculum, upper blade of gcl; lower blade of gcl; s, superficial; d, deep; ml, molecular layer of the dentate; sgz, subgranular zone; so, stratum oriens; pcl, pyramidal cell layer; mf, mossy fiber zone; sr, stratum radiatum; slm, stratum lacunosum-moleculare; scl, subicular cell layer; spl, subicular plexiform layer; solid gray line, hippocampal fissure; dashed gray line, boundary between sr and slm; red line, separation between superficial and deep CA1 pyramidal neurons.

The description of cytoarchitectural traits in vespertilionid and phyllostomid bats follows the classical hippocampal tri-synaptic loop of information processing in the hippocampus (Johnston and Amaral, 1998). The dentate gyrus granule cell layer (gcl) shows the usual densely packed appearance, with an elongated upper (suprapyramidal) blade in the dorsal and intermediate hippocampus (Figures 1A–C, 2A–C). The dorsally short lower (infrapyramidal) blade becomes longer beyond the intermediate level, surpassing the length of the upper blade ventrally (Figures 1D–F, 2D–F). *Myotis nigricans* was the only species in which length differences between blades was not extensive (Figures 1B, E). The hilar polymorphic cell layer (hpcl) is separated from the granule cell layer by a wide cell-poor subgranular zone, which is even wider in phyllostomid (Figure 2) than in vespertilionids bats (Figure 1). In vespertilionid bats, hilar polymorphic neurons are dispersed, whereas in phyllostomid bats, they form a dense band. The CA3 pyramidal neurons are similarly organized in both families, with a dorsal dense band of large pyramidal neurons (Figures 1A–C, 2A–C) becoming loosely arranged ventrally (Figures 1D–F, 2D–F). A tendency for proximally small CA3 pyramidal neurons to become larger distally was observed in some species (see example of *Phyllostomus discolor* in Supplementary Figure 1). Beyond the tip of the mossy fiber zone, the transition from CA3 to CA1 pyramidal neurons is relatively short in phyllostomid bats (Figure 2), in contrast to a more gradual shift seen in vespertilionid bats (Figure 1). This transition zone contains a blend of large and small pyramidal cells, and is likely to correspond to Lorente de Nó’s (1934) CA2. The wide CA1 pyramidal neuron layer is characterized by deep pyramidal neurons dispersing into stratum oriens (Figures 1, 2). Dispersal of CA1 pyramidal cells is most prominent in the tailed tailless bat (*Anoura caudifer*), in which also superficial CA1 pyramids extend much further toward the hippocampal fissure than those in CA3 (Figures 2B, E). Cytoarchitectural arrangements of CA1 vary between species. Superficial CA1 pyramids in *Vespertilio murinus* (Figure 1A) and species of the myotis group are arranged into a compact layer one to two neurons wide. The condensation of superficial pyramids is present at various degrees in all other species, but least prominent in *Desmodus rotundus* (Figures 2C, F) where superficial and deep pyramids appear largely homogenously distributed. Small species-specific variations in the arrangements of deep versus superficial CA1 pyramidal neurons can be seen along the proximodistal and dorsoventral (septotemporal) axis. The transition from CA1 to the subiculum is gradual in all bats. The subiculum itself is small in both families, recognized by a condensation of horizontally oriented neurons towards the alveus. Additionally, the boundary is marked by a more heterogeneous population of neurons, varying in both shape and size, and superficial neurons do not reach as far into stratum radiatum as in CA1 (see for example Figures 1C, F). The presubiculum is marked in both bat families by increased cell density and reduced cell size across all layers.

### 3.2 Calcium binding proteins

The distribution of calcium binding proteins showed many commonalities within the two taxonomic groups, Vespertilionidae and Phyllostomidae, represented in the sample. They are described in the species best representing the two groups, the parti-colored bat (*Vespertilio murinus*) and the pale spear-nosed bat (*Phyllostomus discolor*). Shorter notes provide observations in two additional species from each group *(Myotis nigricans, Pipistrellus pipistrellus, Desmodus rotundus and Anoura caudifer*). Unless noted otherwise, descriptions are applicable to all dorsoventral levels of the hippocampus.

#### 3.2.1 Calretinin

**FIGURE 3:**
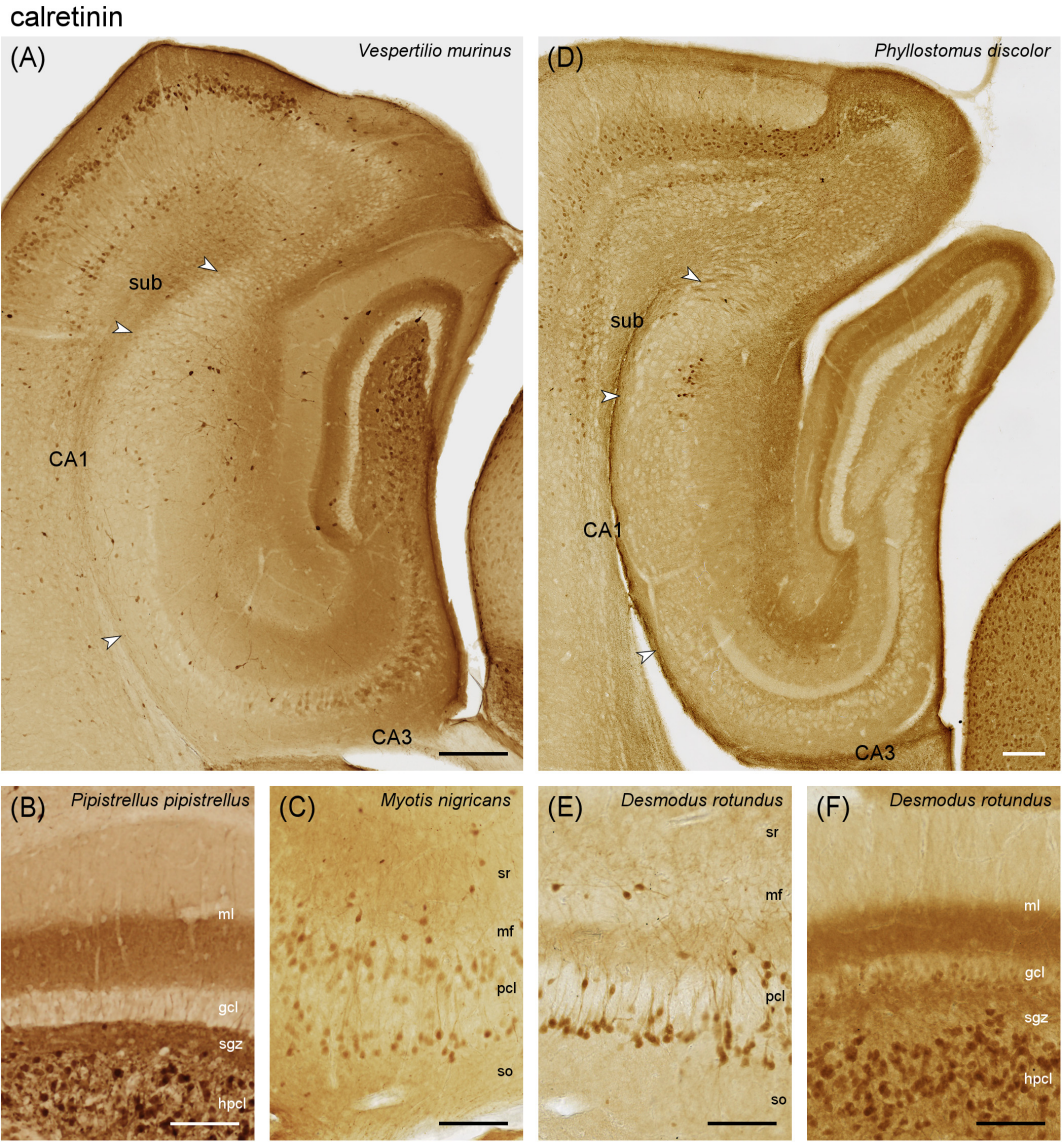
Calretinin immunoreactivity in the vespertilionid **(A–C)** and phyllostomid **(D–F)** bat hippocampus. **(A)** Intermediate hippocampal region of *Vespertilio murinus*. Strong CR+ staining in deep molecular layer of the dentate gyrus, neuronal CR+ staining in hilus, proximal CA3 pyramidal cell layer and interneurons of the CA3 and, more numerous CA1 tiers. Note the strong neuronal CR+ staining in layer ll of the medial entorhinal cortex (MEC). **(B)** Dentate gyrus layers of *Pipistrellus pipistrellus* with stained deep molecular layer and neuronal staining in the hilus. **(C)** In temporal CA3 of *Myotis nigricans*, many deep and superficial CA3 pyramids are CR+, whereas CR+ pyramids are rare in the middle tier. **(D)** Intermediate hippocampal region of *Phyllostomus discolor*. In the dentate gyrus, the middle molecular layer stains CR+. Hilar neurons are weakly CR+. CR+ interneurons are very rare or absent in CA3, CA1, and SUB. A small cluster of proximal superficial subicular neurons is CR+. A CR+ band appears between CA3 pyramidal cell layer and unstained mossy fiber zone. **(E)** Mid-proximodistal CA3 of *Desmodus rotundus*. Deep CA3 pyramidal cells are CR+. A CR+ band similar to that in *Phyllostomus discolor* is visible superficial to the pyramidal cell layer. **(F)** Dentate gyrus layers of *Desmodus rotundus*. A distinct middle molecular layer staining is absent. Some deep granule cell and many neurons of the hpcl are CR+. gcl, granule cell layer hpcl, hilar polymorphic cell layer, mf, mossy fiber zone, ml, molecular layer, sgz, subgranular zone, so, stratum oriens, sr, stratum radiatum, sub, subiculum, pcl, pyramidal cell layer. Scalebars **(A,D)**: 200 μm, **(B,C,E,F)**: 100 μm.

Vespertilionidae – In *Vespertilio murinus*, the deep dentate molecular layer (commissural-associational zone, Figure 3A) was strongly calretinin immunoreactive (CR+). Frequent, large and polymorphic CR+ cells were seen in the hilar polymorphic cell layer (hpcl, Figure 3A, see same characteristics in *P. pipistrellus* in Figure 3B). In addition, some strongly CR+ neurons with interneuronal morphologies are present in the molecular layer and, in the hilus, with some preference for the boundary between granule cell layer and subgranular zone (Figure 3A). Many mainly proximal CA3 pyramidal cells were CR+ (Figure 3A). Their number decreased temporally. In addition, CR+ interneurons were scattered over the other CA3 layers (Figure 3A), increasing temporally. CR+ interneurons appeared more frequent in CA1 than in CA3, and were located preferentially in the deep pyramidal cell layer and around the boundary between stratum lacunosum-moleculare and stratum radiatum (Figure 3A). Like in CA3, interneuronal CR staining increased temporally. The staining pattern continued into the subiculum. A plexus of CR+ coarse, varicose fibers was seen in the subicular plexiform layer (Figure 3A). This general pattern was also seen in *Myotis nigricans* (Figure 3C), and *Pipistrellus pipistrellus* (Figure 3B). In *Myotis nigricans*, CA3 pyramidal and hilar polymorphic neurons stained stronger, but the number of CR+ interneurons was lower, in particular in the dentate gyrus. Temporally, CR+ pyramidal cells were found at depth and superficially in the pyramidal cell layer (Figure 3C), while the middle tier remained unstained. In all three species, we observed strong CR staining in layer II neurons of the medial entorhinal cortex (Figure 3A and Supplementary Figures 3D, E). Notably, this was not accompanied by strong staining in the middle dentate molecular layer (medial perforant path zone in rodents).

Phyllostomidae – In *Phyllostomus discolor*, strong staining was seen in the middle molecular layer (Figure 3D), possibly originating from CR+ neurons in layer II of the temporal medial entorhinal cortex (not yet present at the level illustrated in Figure 3D). A subset of temporal granule cells was weakly CR+ (illustrated for *Desmodus rotundus* in Figure 3F). The deep molecular layer was lightly CR+. CR immunoreactivity was also seen in large, polymorphic neurons of the hpcl (Figure 3D). In contrast to Vespertilionidae, CR+ cells with interneuronal morphologies were very rare (< 1 per section) in the dentate gyrus, CA3, CA1 and subiculum (Figure 3D). In CA3, distal and deep CA3 pyramidal cells were CR+ in the temporal hippocampus. Notably, a CR+ band was seen immediately apical to the pyramidal cell layer of CA3 (Figure 3D, see also Figure 6D). The band was narrow proximally and widened distally. In the stratum lacunosum moleculare of CA3 and CA1, CR immunoreactivity resembled the distribution of medial entorhinal cortex afferents (Figure 3D). A small but distinct group of CR+ pyramidal cells was located superficially in the proximal subicular cell layer (Figure 3D). Staining patterns were very similar in *Anoura caudifer* (lighter and fewer CR+ elements, Supplementary Figure 3C) and the common vampire bat (*Desmodus rotundus*, generally darker and more CR+ elements). Calretinin was not observed in the middle molecular layer of either species (Figure 3F and Supplementary Figure 3C), and the CR+ band superficial to CA3 pyramidal cells was not visible in *Anoura caudifer*. In *Desmodus rotundus*, CR+ deep CA3 pyramidal cells were more frequent than in *Phyllostomus discolor* (Figure 3E), and they occurred along the entire dorsoventral axis. Small bipolar immunoreactive interneurons (rare in most bats of our sample but common in other mammal species) were scattered throughout the layers of CA1 and CA3 in *Desmodus rotundus*, except for stratum lacunosum-moleculare. In addition to a large group of immunoreactive cells in the superficial proximal subicular cell layer, a few immunoreactive CA1 pyramids were seen adjacent to the subicular group.

#### 3.2.2 Calbindin

**FIGURE 4:**
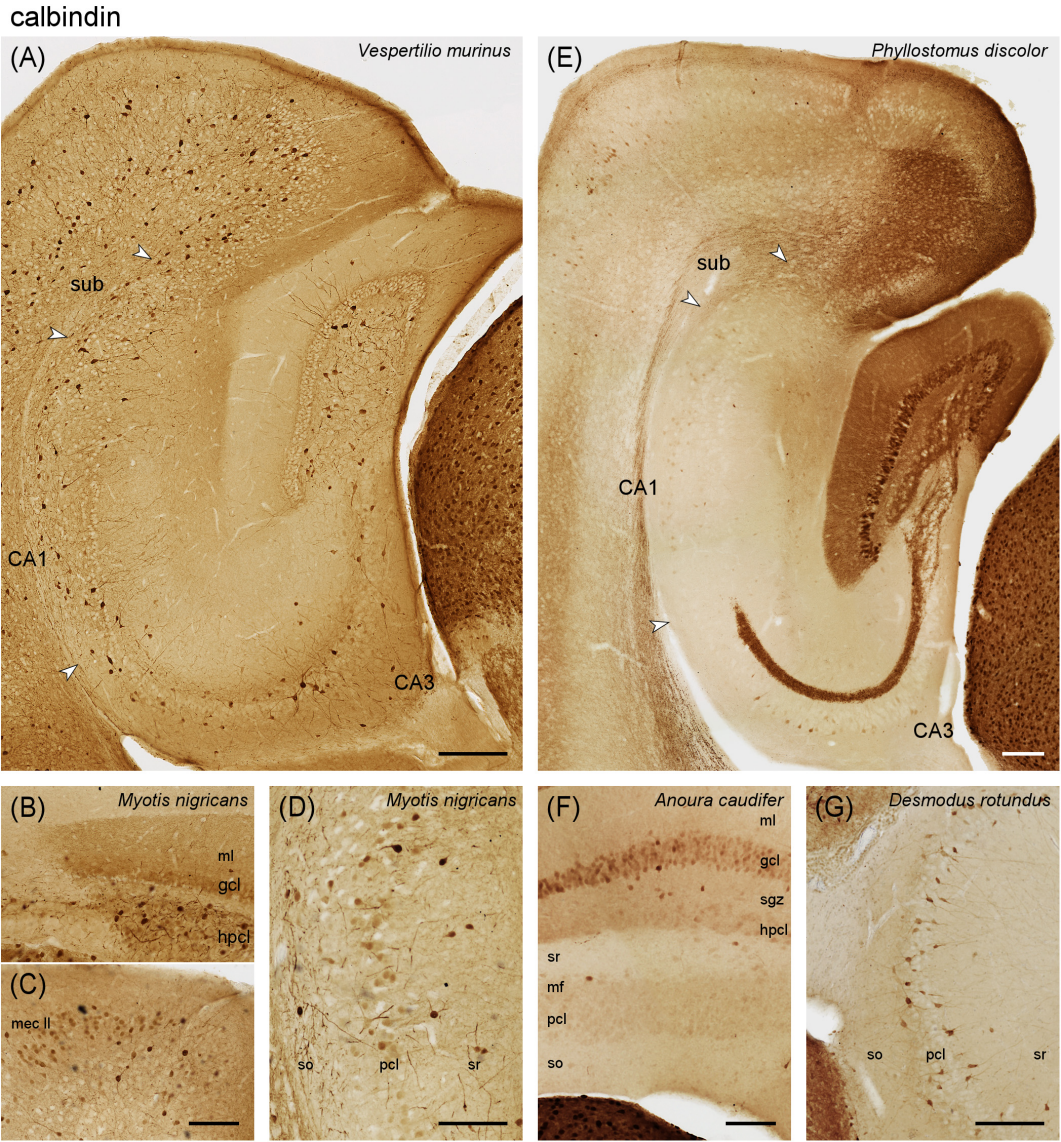
Calbindin immunoreactivity in the vespertilionid **(A–D)** and phyllostomid **(E–G)** bat hippocampus. **(A)** Intermediate hippocampal region of *Vespertilio murinus*. **(B)** Dentate gyrus and adjacent CA3 of *Myotis nigricans*. Pale staining is seen in dentate granule cells and molecular layer, which may relate to **(C)** calbindin expression in entorhinal layer II neurons. **(D)** Some superficial pyramidal cells in CA1 of *Myotis nigricans* express calbindin. **(E)** Intermediate hippocampus of *Phyllostomus discolor* **(F)** Dentate gyrus and adjacent CA3 of *Anoura caudifer*. Generally weak calbindin expression is seen in granule cells and the mossy fiber zone. **(G)** Calbindin expression in all hippocampal regions, here CA3, of *Desmodus rotundus* resembles the pattern seen in vespertilionid bats. gcl, granule cell layer hpcl, hilar polymorphic cell layer, mf, mossy fiber zone, mec II, medial entorhinal cortex layer II, ml, molecular layer, sgz, subgranular zone, so, stratum oriens, sr, stratum radiatum, sub, subiculum, pcl, pyramidal cell layer. Scale bars **(A,E)**: 200 μm, **(B–D,F,G)**: 100 μm.

Vespertilionidae – In *Vespertilio murinus*, calbindin immunoreactive (Calb+) neurons with interneuronal morphologies were found in the subgranular and hilar polymorphic cell layers (Figure 4A). Granule cells and, consequently, the mossy fiber zone of CA3 were only very weakly Calb+. Calb+ neurons with interneuronal morphologies were associated with the pyramidal cell layer of CA3 and the deep pyramidal cell layer of CA1 (Figure 4A). The number of Calb+ neurons increased slightly in the deep subicular cell layer. In *Myotis nigricans*, granule cell and mossy fiber staining was stronger than in *Vespertilio murinus*, but still weak. In addition, moderate staining was seen in the outer two-thirds of the dentate molecular layer (Figure 4B). Calb+ entorhinal layer II neurons were only seen in the medial part of the medial entorhinal cortex (Figure 4C). A subset of superficial CA1 pyramidal cells was weakly Calb+ (Figure 4D). Calb+ subicular neurons were less frequent than in *Vespertilio murinus*. Most hippocampal layers of *Pipistrellus pipistrellus* stain indistinguishable from *Vespertilio murinus*, with the exception of the subiculum, in which Calb+ neurons were much fewer than in adjacent CA1 (Supplementary Figure 3F).

Phyllostomidae – In *Phyllostomus discolor*, the vast majority of dentate granule cell were Calb+ (Figure 4E). The subgranular zone showed moderate staining, which increased over the hpcl and continued with equal intensity into the mossy fiber zone of CA3 (Figure 4E). There was an unstained gap between a Calb+ mossy fiber zone and the pyramidal cell layer (Figure 4E, see also Figures 6B–D). Similar to the CR+ band, it was narrow proximally and widened distally. A few, weakly Calb+ neurons of pyramidal morphology were scattered within the deep distal pyramidal cell layer of CA3 and in the middle proximal cell layer of the subiculum (Figure 4E). Immunoreactive cells with clear interneuronal morphologies were very rare in the hippocampus (< 1/region and section). This was also observed in adjacent cortices even though layer II/III projection neurons were Calb+, which can be observed in many species, and strongly Calb+ neurons were present in other brain division (Figure 4E). Staining was much weaker in *Anoura caudifer* (Figure 4F), but the elements identified in *Phyllostomus discolor* were present. Clear Calb+ interneurons were again very rare. *Desmodus rotundus* deviated strongly from the pattern observed in the other two phyllostomatid bats, but instead resembled the pattern seen in vespertilionid bats (Figure 4G). Dentate granule cells and the mossy fiber zone remained very weakly stained. Instead, frequent Calb+ interneurons were scattered evenly throughout and adjacent to the cell layers of CA3 (Figure 4F), CA1 and the subiculum. The staining pattern of *Desmodus rotundus* and vespertilionid bats strongly resembled the distribution of parvalbumin. Mouse sections run in the same batches invariably showed the typical pattern described by others, including strong staining of granule cells and mossy fibers that in our sample of bats is only seen in *Phyllostomus discolor*.

#### 3.2.3 Parvalbumin

**FIGURE 5:**
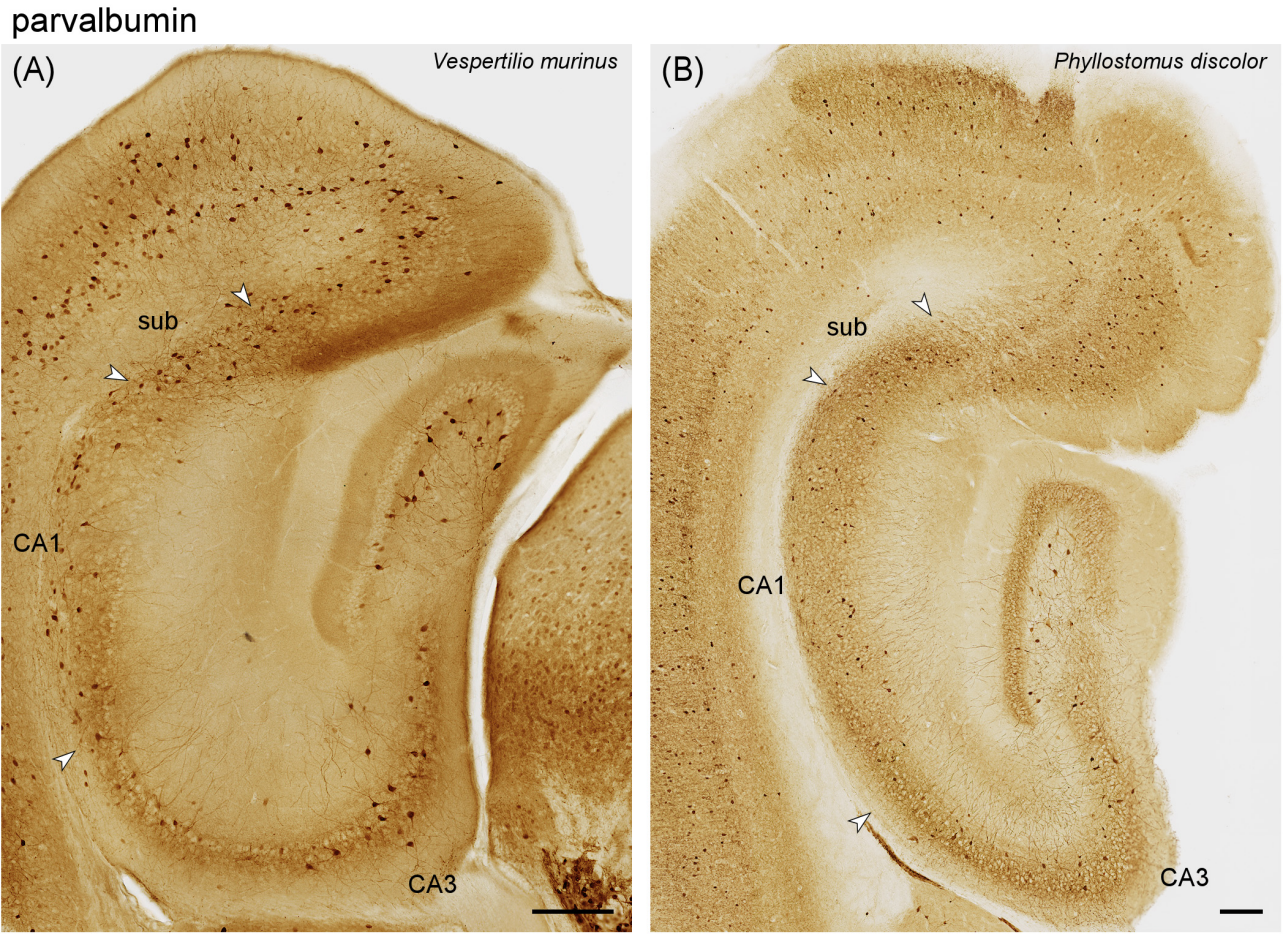
Parvalbumin immunoreactivity in a vespertilionid **(A)** and phyllostomid **(B)** bat hippocampus. **(A)** Intermediate hippocampal region of *Vespertilio murinus*. **(B)** Hippocampal region of *Phyllostous discolor*. This section is located slightly more dorsal than **(A)** because the staining pattern in the matched section did not reflect the typical distribution of parvalbumin in this species. Scalebars 200 μm.

Vespertilionidae – In *Vespertilio murinus*, the deep molecular layer was weakly parvalbumin positive, even though there was no staining indicative of mossy cells in the hpcl (Figure 5A). Interneurons were frequent in both the subgranular zone and the hpcl (Figure 5A), and they included pyramidal basket-like neurons at the deep border of the granule cell layer. The distribution seen in the hpcl continued into the pyramidal cell layer of CA3 and CA1 (Figure 5A). Compared to CA1, the number of parvalbumin immunoreactive neurons dropped slightly in the subicular cell layer. Throughout the dentate and hippocampus, fine granular staining was found throughout the cell layers (Figure 5A). Parvalbumin-positive interneurons were also common in stratum radiatum of CA3 and stratum oriens in CA1 (Figure 5A). An increase at the CA1–CA3 boundary, a characteristic of the parvalbumin distribution in many species, was not seen (Figures 5A, B). In *Myotis nigricans*, the general staining pattern resembled that seen in *Vespertilio murinus*, but the number of immunoreactive neurons appeared markedly lower (Supplementary Figure 3A). The parvalbumin staining pattern of *Pipistrellus pipistrellus* is very similar to that of *Vespertilio murinus* (Supplementary Figure 3B).

Phyllostomidae – The staining pattern observed in all three phyllostomid bats largely resembles that of the vespertilionid bats. Staining is a little stronger in *Phyllostomus discolor* (Figure 5B) than in *Vespertilio murinus* (Figure 5A) and the two other phyllostomid bats. The density of stained elements remained unchanged across the CA1-CA3 boundary in *Phyllostomus discolor* (Figure 5B) and in other phyllostomid species.

### 3.3 Timm staining in phyllostomid bats

**FIGURE 6:**
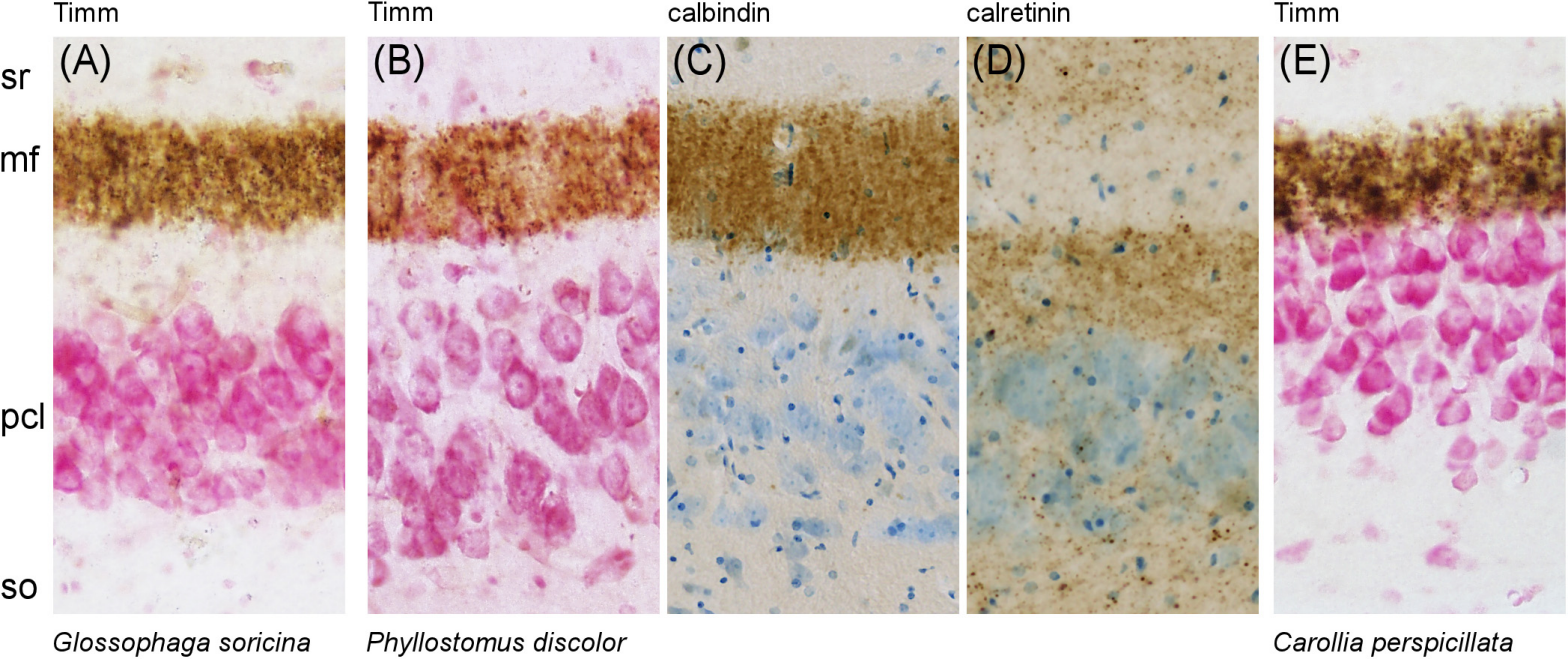
Species-specific gap in the CA3 region of phyllostomid bats. An unusual separation between the Timm- and calbindin-positive mossy fiber zone and the CA3 pyramidal cell layer (pcl) is observed in phyllostomid bats. **(A)** In *Glossophaga soricina*, the gap is pronounced. (B–D) In *Phyllostomus discolor*, the gap is moderate but clearly visible: **(B)** Timm and **(C)** calbindin staining show a separation between the mossy fiber terminals and the CA3 pcl, while **(D)** calretinin staining reveals this gap is filled with a band of punctate calretinin-positive fibers. **(E)** In contrast, *Carollia perspicillata* shows no such gap, presenting the typical mammalian pattern with mossy fibers stained with Timm closely adjacent to the CA3 pcl.

To explore the unusual staining patterns of calbindin and calretinin in the CA3 region of phyllostomids, we evaluated mossy fiber stains in *Glossophaga soricina* (Figure 6A), *Phyllostomus discolor* (Figure 6B), and *Carollia perspicillata* (Figure 6E). In *Phyllostomus discolor*, Timm-positive staining of zinc-containing mossy fiber boutons in the proximal CA3 were adjacent to the CA3 pyramidal cell layer, while in the distal CA3 (Figure 6B) the zinc-positive band of mossy fibers separated from the pyramidal layer. The same proximal to distal pattern of change was observed for mossy fiber zone calbindin immunoreactivity (Figure 6C). The zinc- and calbindin-negative suprapyramidal area was in size and distribution similar to the calretinin-positive plexus seen in this species (Figure 6D). This gap between zinc- and calbindin-positive mossy fiber zone was more pronounced in *Glossophaga soricina* (Figure 6A) than in *Phyllostomus discolor* (Figures 6B, C). The gap was absent in *Carollia perspicillata* (Figure 6E), where the Timm-stained mossy fiber zone borders the CA3 pyramidal layer as typical in other mammals.

### 3.4 Hippocampal neuron numbers in bats

**FIGURE 7:**
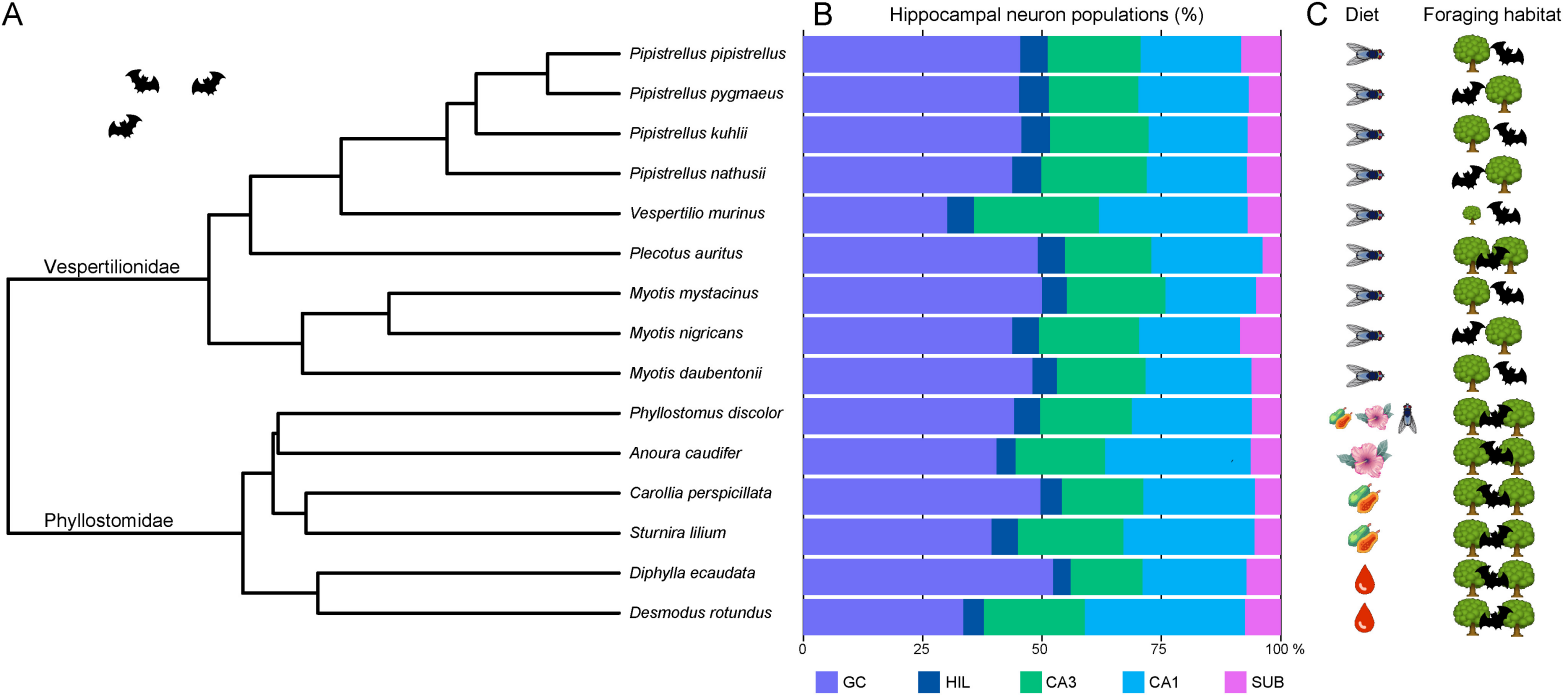
Phylogenetic tree, hippocampal neurons and ecological factors in bats. **(A)** The 15 bat species are shown in a rooted phylogenetic tree extracted from Álvarez-Carretero et al. (2022). **(B)** Relative size of hippocampal neuron populations in percent reveals a rather uniform cellular composition of the hippocampus in insect-eating vespertilionids. Only *Vespertilio murinus* is separated by its markedly smaller granule cell (GC) population. Phyllostomids are more variable in their relative neuron population sizes than vespertilionids, differing even between closely related species pairs. Typical for all bats is the remarkably small subicular (SUB) population, accounting for only ∼6.6% of all hippocampal principal neurons. **(C)** Ecological factors such as diet and foraging habitat [adapted from Denzinger and Schnitzler (2013)] visualize that all vespertilionid species of our sample are insectivores and most are edge space aerial foragers, except *Vespertilio murinus* (open space aerial forager) and *Plecotus auritus* (narrow space gleaner). Phyllostomids are more variable in terms of diet, ranging from omnivorous, nectivorous and frugivorous to the hematophagous vampire bats, but all are narrow space gleaners. GC, granule cells; HIL, hilar neurons; CA3 resp. CA1 pyramidal neurons; SUB, subicular neurons. (Pictograms were taken from Illustrator symbols or icons8.com).

Absolute numbers of hippocampal neurons (Table 2) varied considerably between species, as did body and brain sizes (Table 1, see also Supplementary Figure 2). For comparisons, neuron numbers of each principal neuron populations were therefore presented in percentage of total hippocampal neuron numbers (Figure 7). Within vespertilionids (Figure 7A), the proportion of each neuron population within the hippocampus was relatively uniform, with the exception of *Vespertilio murinus* where the percentage of granule cells was smaller in favor of more CA1 pyramidal neurons (Figure 7B). Proportions of neuronal populations were more variable in phyllostomids, even between closely related species. Granule cell numbers differed by 10% between the two frugivore bats (*Carollia perspicillata* and *Sturnira lilium*), and by 18% between the two vampire bats (*Diphylla ecaudata* and *Desmodus rotundus*). The proportion of subicular neurons was small in all bats.

**TABLE 2:** Total cell number estimates for the five hippocampal neuron populations.

Vesperti-lionidae *n* (by sex)	*Myotis daubentonii* 3 (f:2;m:1)	*Myotis mystacinus* 2 (m:2)	*Myotis nigricans* 2 (m:2)	*Pipistrellus kuhlii* 4 (f:1; m:3)	*Pipistrellus nathusii* 4 (m:4)	*Pipistrellus pipistrellus* 5 (f:1; m:4)	*Pipistrellus pygmaeus* 1 (f:1)	*Plecotus auritus* 1 (f:1)	*Vespertilio murinus* 1 (m:1)
**Granule cells**									
Mean	238,499	219,149	148,152	195,591	201,518	164,566	180,049	292,982	115,915
SD	22,135	33,303	31,475	42,220	30,848	41,402	–	–	–
Mean CE (m = 0)	0.1	0.07	0.08	0.08	0.09	0.09	0.1	0.08	0.1
**Hilar neurons**									
Mean	25,781	22,530	19,002	25,939	27,889	20,192	24,847	33,916	21,539
SD	1,878	1,190	700	7227	2,613	2,510	–	–	–
Mean CE (m = 0)	0.09	0.07	0.07	0.04	0.06	0.06	0.05	0.08	0.06
**CA3 pyramidal neurons**									
Mean	92,316	90,460	70,163	87,943	102,179	68,538	74,359	108,249	100,487
SD	8,295	5,563	6,975	15,550	18,842	5,991	–	–	–
Mean CE (m = 0)	0.09	0.07	0.07	0.06	0.07	0.07	0.09	0.07	0.07
**CA1 pyramidal neurons**									
Mean	109,686	82,457	71,206	87,654	95,869	75,425	92,326	139,135	118,123
SD	23,389	3,980	2,794	17,752	9,582	14,195	–	–	–
Mean CE (m = 0)	0.09	0.06	0.07	0.096	0.06	0.07	0.1	0.08	0.08
**Subicular neurons**									
Mean	30,432	22,337	28,345	29,516	32,528	28,986	26,386	21,469	30,308
SD	2,319	1,201	3,022	6,716	7,844	1,792	–	–	–
Mean CE (m = 0)	0.09	0.08	0.07	0.08	0.08	0.08	0.1	0.11	0.08
Phyllostomidae *n* (by sex)		*Anoura caudifer* 4 (m:4)	*Carollia perspicillata* 4 (f:2; m:2)	*Desmodus rotundus* 3 (f:3)		*Diphylla ecaudata* 4 (f:1; m:3)	*Phyllostomus discolor* 5 (f:1; m:4)		*Sturnira lilium* 3 (m:3)
**Granule cells**									
Mean		486,137	606,367	321,307		582,337	606,194		493,066
SD		31,564	119,093	17,814		198,698	77,666		150,102
Mean CE (m = 0)		0.1	0.09	0.08		0.06	0.08		0.09
**Hilar neurons**									
Mean		48,152	54,452	41,379		39,908	74,867		67,291
SD		5,595	9,937	3,648		7,046	13,169		5,602
Mean CE (m = 0)		0.08	0.11	0.09		0.08	0.07		0.09
**CA3 pyramidal neurons**									
Mean		225,902	204,652	202,691		162,374	262,170		268,842
SD		14,054	14,356	40,648		36,755	31,155		26,566
Mean CE (m = 0)		0.07	0.06	0.07		0.08	0.06		0.07
**CA1 pyramidal neurons**									
Mean		367,350	282,532	320,894		232,067	346,509		338,334
SD		51,655	31,209	19,946		21,590	51,837		90,531
Mean CE (m = 0)		0.06	0.06	0.06		0.07	0.06		0.07
**Subicular neurons**									
Mean		75,737	65,398	71,309		77,521	81,999		67,788
SD		8,594	15,016	7,437		8,594	11,206		19,999
Mean CE (m = 0)		0.09	0.08	0.08		0.09	0.09		0.1

Numbers are given in mean and standard deviation (SD); precision of estimates (CE, Coefficient of Error, for smoothness factor m = 0) are reported. Neuron counts were obtained using the optical fractionator method in Nissl-stained sections.

**FIGURE 8:**
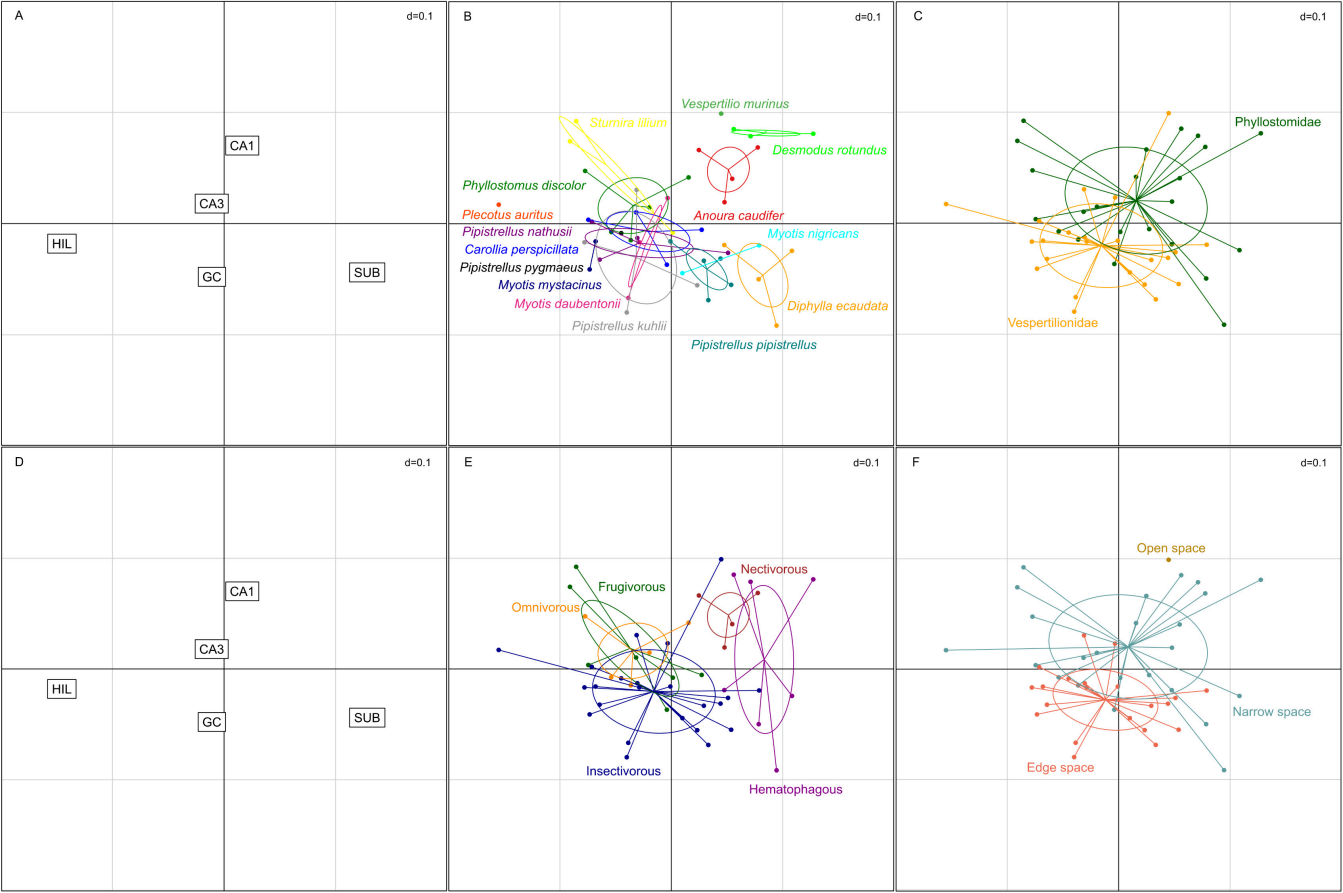
Correspondence analysis of hippocampal neuron populations in echolocating bats. **(A,D)** The two smallest neuron populations, hilus (HIL) and subiculum (SUB), were strongest differentiators on the first axis, while CA1 pyramidal neurons and granule cells (GC) were driving separation along the second axis. CA3 pyramidal neurons contributed least to the differentiation. **(B)** Increased weight for HIL and GC neurons clustered many species to the lower left quadrant, yet some species formed distinct clouds. **(C)** Separation by family revealed increased weight to the output side of the hippocampus (SUB and CA1) in phyllostomids, while vespertilionids were dominated by increased weight of the input side of the hippocampus (GC and HIL), however considerable overlap was apparent between the two families. **(E)** Preferred diet separated the hematophagous bats and the nectivorous bat from the frugivorous, omnivorous and insect eating predatory bats along the first axis. **(F)** Foraging habitats separated the species in a similar way as families, with the exception of the open space foraging *Vespertilio murinus* which does not cluster with the narrow or edge space foragers.

For the correspondence analysis (Figure 8), estimated total neuron numbers were z-transformed. The two axes in Figure 8 represent 85.5% of the variance in the data (1st factor, x-axis: 52.6%, 2nd factor, y-axis: 32.9%). Hilar and subicular neurons, the smallest neuron populations, caused the largest separation between species and dominated the first axis, while the second axis was dominated by granule cells and CA1 pyramidal neurons (Figure 8A). CA3 pyramidal neurons did not have much differentiating power. Species distribution (Figure 8B) already indicated that there is considerable overlap between the two families (Figure 8C), but separation of the two clusters suggested an increased emphasis on input neuron populations of the hippocampus (granule and hilar neurons) in vespertilionids, while the output side (CA1 pyramidal and subicular neurons) appeared more dominant in phyllostomids. Within the family of phyllostomid bats, preferred diet (Figure 8E) segregated the omnivore and frugivore bats (increased weight on HIL) from the nectivorous and hematophagous vampire bats (increased weight on SUB) along the first axis of the correspondence analysis. Foraging habitat (Figure 8F) was nearly congruent with families, again indicating a tendency to an input dominant hippocampus in edge space foragers, while narrow space foragers had more weight in output neuron populations of the hippocampus. The only open space forager (*Vespertilio murinus*) was separated from all other species in this analysis due to its increased weight on CA1.

## 4 Discussion

### 4.1 General cytoarchitectural characteristics in the bat hippocampus

We focus here on two features in the hippocampal cytoarchitecture of small echolocating bats. The first observation is the length difference of the upper and lower blade of the granule cell layer that shifts along the dorsoventral axis. Anatomical asymmetry in the dentate gyrus is not uncommon and has been described in detail for rats early on [reviewed by Amaral et al. (2007)], length difference between dentate blades in rodents appear however on a smaller range than what we observed in bats. In rodents, the upper and lower blade differ in their morphological (Gallitano et al., 2016), physiological (Mishra and Narayanan, 2020; Strauch et al., 2025) and functional (Chawla et al., 2005; Ramírez-Amaya et al., 2005; Satvat et al., 2011; Schmidt et al., 2012) characteristics. Whether these properties extend to the bat dentate gyrus too is currently not known, but one might wonder about the advantage of a prominent upper blade in the dorsal and intermediate dentate gyrus. In rats, granule cells in the upper blade are more activated during spatial tasks than those in the lower blade (Chawla et al., 2005; Ramírez-Amaya et al., 2005; Schmidt et al., 2012), thus a prominently elongated upper blade as seen in bats might increase precise spatial processing, which is the domain of the dorsal hippocampus (Strange et al., 2014). Specific functional attributes to the lower blade are, to our knowledge, currently unknown. Spatial exploration induces more Arc-expression in the upper than lower blade in the dorsal dentate gyrus, however the lower blade responds to the experimental exposure with a delayed and transient Arc-expression (Ramírez-Amaya et al., 2005). We found in bats that the lower blade is prominently elongated in the most ventral part of the dentate gyrus, a region of the hippocampus traditionally associated with emotional responses (Strange et al., 2014). To elucidate the specific function of lower blade granule cells, one may have to venture into behavioral tests for fear, motivation or reward, and small echolocating bats with their prominent lower blade ventrally might become especially suitable models to do so.

The second feature is the radial expansion of the CA1 pyramidal cell layer in all bats presented here. The distribution of the superficial and deep CA1 pyramidal cells in bats is reminiscent of that seen in primates including humans (Lorente de Nó, 1934; Slomianka et al., 2011). Deep and superficial CA1 pyramidal cells differ in their development, gene expression profile and connectivity (Slomianka et al., 2011; Valero and de la Prida, 2018; Cembrowski and Spruston, 2019) and process different information within the hippocampal circuits (Geiller et al., 2017; Soltesz and Losonczy, 2018; Harvey et al., 2023). In the context of the bat CA1, findings by Sharif et al. (2021) in mice might be most interesting, as they report more place cell activity in cue-rich environment in deep CA1 pyramids, while a cue-poor environment is linked to increased neuronal activity in superficial CA1 pyramids. Most bats commute relatively long distances to their feeding grounds, and one can assume this behavior corresponds to a distinct shift between cue-poor navigation in open space and cue-rich navigation once animals start feeding. Previous studies have confirmed CA1 place cells in large fruit bats (Yartsev and Ulanovsky, 2013) and echolocating bats (Ulanovsky and Moss, 2007), without differentiating between deep and superficial cells. The well-differentiated CA1 in bats would be convenient to explore flexible navigation in response to spatial maps of different scale in a natural environment.

### 4.2 Calcium binding proteins

With the verspertillionid and phyllostomid small echolocating bats, some information is now available for the second most speciose group of mammals. Together with the information already available from many other clades, it permits not only to (yet again) point out species differences and similarities, but also to outline some basic ideas about how the distribution of calcium-binding proteins may serve in understanding hippocampal function.

#### 4.2.1 Calretinin

Like calbindin (see below), the hippocampal expression of calretinin is highly species variable (Murakawa and Kosaka, 1999). In laboratory mice, it may serve as a marker for dentate mossy cells in the temporal hippocampus (Fujise et al., 1998), while it is absent or only found in the temporal extreme of other species (Seress et al., 2008; Maliković et al., 2023; Maliković et al., 2024). Bats add to the spectrum of variability by the presence of CR + mossy cells throughout the dorsoventral extent of the dentate gyrus. Calretinin is not observed in hippocampal pyramidal cells of laboratory mice or rats (Gulyás et al., 1992; Liu et al., 1996), but has been observed in subsets of CA3 pyramids in other species such as spiny rat (Fabene et al., 2001), banded mongoose (Pillay et al., 2021) and wild boar (Maliković et al., 2023). Calretinin expression in CA3 pyramids is generally more extensive in bats. In particular in *Myotis nigricans*, temporal CA3 pyramidal calretinin expression resembles the highly differentiated CA1 calbindin expression in, e.g., dog (Hof et al., 1996) or tree pangolin (Imam et al., 2019).

#### 4.2.2 Calbindin

While calbindin was found to be a marker expressed by granule cells and in the mossy fiber zone of many mammals (Rami et al., 1987; Hof et al., 1996; Amrein et al., 2014; Maliković et al., 2024), the staining of other elements was found to be variable between species. The distribution of calbindin in the hippocampi of small echolocating bats again confirmed and extended these observations. Calbindin may be very weak or even absent from granule cells and mossy fibers and showed large differences even between taxonomically close species, as exemplified by *Desmodus rotundus* when compared to two other phyllostomid species. The histoarchitectural differentiation of CA1 is, in contrast to other species (Hof et al., 1996; Imam et al., 2019), not reflected in a corresponding differentiation by calbindin. Instead, a few Calb+ CA3 pyramidal cells were found in CA3 of *Phyllostomus discolor*, a trait previously only seen in the naked mole-rat (Amrein et al., 2014). Also notable is the scarcity of Calb+ interneurons, including those in the deep stratum oriens, in phyllostomid bats and tree pangolin (Imam et al., 2019) when compared to laboratory rodents (Sloviter, 1989; Celio, 1990; Jinno and Kosaka, 2006) or primates (Seress et al., 1991). Even though very rare in most layers, at least the deep stratum oriens Calb+ interneurons were a prominent feature of the sengi hippocampus (Slomianka et al., 2013). While the absence of a marker does not necessarily mean that the cell types usually marked are absent, at least calbindin seems not necessary for their normal function in many species.

Clade or species-specific marker expression may serve, likely along a continuum, two distinct purposes. A marker may be expressed to ensure consistent function despite changes of other physiological or anatomical network characteristics, i.e., marker expression is a network-emergent, homeostatic property. Alternatively, marker expression may alter functional properties to mediate species-specific demands on hippocampal information processing. It would be interesting to see were along this continuum the expressions of calretinin and calbindin are placed. However, the specific functional properties resulting from expression of calbindin (Molinari et al., 1996; Jouvenceau et al., 1999; Pasti et al., 1999; Jouvenceau et al., 2002; Li et al., 2017; Schwaller, 2020) or calretinin (Gurden et al., 1998; Todkar et al., 2012; Schwaller, 2020) may be of limited value in the development of translatable concepts of hippocampal function. In this context, the prime value of markers like calbindin and calretinin lies more in the anatomical definition of neuron populations that can react, by way of marker expression, in unison rather than in the specific physiological consequences of expression.

#### 4.2.3 Parvalbumin

Similar to previous assessments (Maliković et al., 2024), we again found parvalbumin expression to be rather consistent, and the pattern seen in bats is very similar to that observed in other species. A small deviation is the lack of an increase or even a decrease in the apparent number of Parv+ elements at the CA1–CA3 boundary in bats. In contrast to calbindin and calretinin, the consistency of parvalbumin expression in similar neurons across species would suggest that it is not only the circuit defined by the marker that is important to understand hippocampal function (Murray et al., 2011; Donato et al., 2013; Le Roux et al., 2013; Stark et al., 2014; Amilhon et al., 2015; Donato et al., 2015; Karunakaran et al., 2016; Aery Jones et al., 2021), but also the (patho-) physiological consequences of parvalbumin expression within the circuit (Vreugdenhil et al., 2003; Lucas et al., 2010; Filice et al., 2016; Filice et al., 2020; Schwaller, 2020). Even though comparative data are fewer, the same argument may be applied to cholecystokinin, which shows rather consistent interneuronal distributions across species (Gall, 1990; Holm et al., 1993) and which is used as a marker to define inhibitory circuitry complementary to that of Parv+ neurons (Krook-Magnuson et al., 2012; Valero and de la Prida, 2018).

### 4.3 The gap

A highly unusual feature revealed by the distribution of calbindin, calretinin and the Timm stain was the gap between the superficial boundary of the pyramidal cell layer and the zone staining for the classical mossy fiber markers calbindin and zinc in phyllostomids. Tract tracing studies will be needed to resolve the cause of the gap. We considered two possibilities. First, extra- or intrahippocampal CR+ afferents may intervene between pyramidal cells and the mossy fiber zone. Although a likely source does not come to mind, the gap itself is such an unlikely feature that the possibility should not be dismissed. Afferents important enough to displace the mossy boutons from close to the somal center would tell us much about hippocampal information processing in these species. A second possibility is the presence of neurochemically distinct granule cell and/or mossy bouton populations that segregate radially where the gap is visible. A subset of temporal granule cells was CR+, and calbindin was absent from granule cells and mossy fibers in the phyllostomid *Desmodus rotundus* and unusually weak in verspertilionid species. Other species show a heterogenous (Cavegn et al., 2013), sometimes laminar (Slomianka et al., 2013) expression of calbindin in granule cells, i.e., calbindin can be absent or undetectable in (a subset) of granule cells and mossy fiber boutons. Also, a subset of rat mossy boutons did not express the transporter ZnT3 (Rekart and Routtenberg, 2010) responsible for vesicular zinc loading and, consequently, the Timm staining (Cole et al., 1999). Although the two bouton types did not segregate radially in rats, they occurred more frequently in distal CA3 [Figure 3 in Rekart and Routtenberg (2010)], where the gap is widest in bats. The concept of segregated information streams across subsets of CA1 pyramidal cells has become well-established (Cid et al., 2021; Sharif et al., 2021; Ding et al., 2022; Huszár et al., 2022) and evidence is accruing also for CA3 (Slomianka et al., 2011; Marissal et al., 2012; Balleza-Tapia et al., 2022). But streams were originally suggested to apply already to the dentate granule cells (Deguchi et al., 2011), which is supported by a (often birthdate-related) diversity of dentate granule in gene expression and physiology (Shah et al., 2016; Imura et al., 2019; Save et al., 2019; Erwin et al., 2020). Neurochemical diversity and spatial segregation of mossy boutons in phyllostomid bats would provide an early anatomically defined window into hippocampal information streams. In general, the histoarchitectural differentiation of CA1 and the prominent definition of subsets of CA3 cells by calretinin point toward a stronger definition of such streams in small echolocating bats than in many other species. Given that phyllostomid bats are amenable to laboratory colony breeding, they harbor the potential to gain insights into hippocampal function similar to those that were gained from large fruit-eating bats.

### 4.4 The quantitative neuronal makeup of the bat hippocampus

Compared to a diverse sample of mammals (van Dijk et al., 2016), the overall quantitative makeup of hippocampal neuronal populations in echolocating bats shows a mixture of known and unique features. The relatively small granule cell population (on average 45%) was seen in rodents before, while the large hilar neuron population (5%) does not fit the rodent trait. In bats, the potentially stronger feedback control by many hilar neurons on relatively few granule cells may strengthen pattern separation in the dentate gyrus via a gate or filter function [reviewed by Borzello et al. (2023)]. The well-developed CA3 (20%) is a feature observed in mole-rats too, while many CA1 pyramidal neuron (24% in bats) is not a common feature in rodents, but was seen in dog and human (van Dijk et al., 2016), thus, CA1 pyramidal neurons are not only less densely packed in echolocating bats than in many other mammals, they are also quite numerous. Quantitatively, there is no indication that the loose arrangement of CA1 pyramidal neurons as seen in bats, humans and other primates might be a consequence of high numbers of CA1 pyramids within the hippocampal circuitry. While humans do have a high percentage (33%) of CA1 pyramidal neurons, other primates have less than bats (Rhesus monkey 13%, marmoset 17%, van Dijk et al., 2016). Drivers for the loose arrangement of CA1 pyramidal neurons are, to our knowledge, unknown. Striking in comparison to other species such as rodents (on average 12%) or humans (13%) is the small subicular neuron population in bats (7%). The function of the subiculum within the hippocampal network has been extended from a mere relay unit for information transfer between the hippocampus and cortical and subcortical target areas to a unique role for integrating and compressing spatial information of large, complex environments [reviewed by Matsumoto et al. (2019)], with specialized cells such as boundary vector cells (Lever et al., 2009) and vector trace cells (Poulter et al., 2021) responding to objects, boundaries and cues. In this study, we present the subicular neuron population as a homogenous entity. Yet, at least two types of pyramidal cells with different electrophysiological properties and spatial distribution have been described (Ishihara and Fukuda, 2016), neurons in the subiculum also segregate along the proximo-distal axis, e.g., vector trace cells are nearly exclusively found in the distal subiculum of rats (Poulter et al., 2021). How bats navigate successfully in a three-dimensional environment with relatively few subicular neurons still needs to be explored, further investigations would be required to verify if the bat-specific small subiculum stems from a global reduction, or a reduction of specific cell types and/or subregions.

How are ecological factors such as foraging habitat and diet reflected in the neuronal composition of the hippocampus? In the present species sample, we found not a clear separation between edge space and narrow space foragers in terms of hippocampal neuronal composition, which stands in contrast to correlations between hippocampal volume and foraging habitat reported before (Baron et al., 1996b; Safi and Dechmann, 2005). In our analysis, a similar distribution in the correspondence analysis is however seen if bats are grouped by family, thus, space use cannot be separated from phylogenetic traits in the numerical composition of hippocampal neuron numbers in this sample of bats. Diet preferences however did separate phyllostomid bats in their hippocampal cellular composition, which was not found on hippocampal volumetric analysis in this family (Ratcliffe, 2009). The sample of bat species presented here is relatively small, and *Vespertilio murinus* with a unique ecological trait already indicate that the inclusion of more species might reveal structure-function associations between hippocampal neuron numbers and ecological factors that we cannot see at present. Yet, our study presents the first quantitative data set of the bat hippocampus at circuit level providing ample evidence of unique and interesting hippocampal features, and we believe this report will help guiding future studies into this large group of mammals.

## Data Availability

The original contributions presented in this study are included in this article/Supplementary material, further inquiries can be directed to the corresponding author.

## References

[B1] Aery Jones E. A. Rao A. Zilberter M. Djukic B. Bant J. S. Gillespie A. K. (2021). Dentate gyrus and CA3 GABAergic interneurons bidirectionally modulate signatures of internal and external drive to CA1. *Cell Rep.* 37:110159. 10.1016/j.celrep.2021.110159 34965435 PMC9069800

[B2] Álvarez-Carretero S. Tamuri A. U. Battini M. Nascimento F. F. Carlisle E. Asher R. J. (2022). A species-level timeline of mammal evolution integrating phylogenomic data. *Nature* 602 263–267. 10.1038/s41586-021-04341-1 34937052

[B3] Amaral D. G. Scharfman H. E. Lavenex P. (2007). The dentate gyrus: Fundamental neuroanatomical organization (dentate gyrus for dummies). *Prog. Brain Res.* 163 3–22. 10.1016/S0079-6123(07)63001-5 17765709 PMC2492885

[B4] Amilhon B. Huh C. Y. Manseau F. Ducharme G. Nichol H. Adamantidis A. (2015). Parvalbumin interneurons of hippocampus tune population activity at theta frequency. *Neuron* 86 1277–1289. 10.1016/j.neuron.2015.05.027 26050044

[B5] Amrein I. Becker A. S. Engler S. Huang S.-H. Müller J. Slomianka L. (2014). Adult neurogenesis and its anatomical context in the hippocampus of three mole-rat species. *Front. Neuroanat.* 8:39. 10.3389/fnana.2014.00039 24904308 PMC4033039

[B6] Amrein I. Dechmann D. K. Winter Y. Lipp H. P. (2007). Absent or low rate of adult neurogenesis in the hippocampus of bats (Chiroptera). *PLoS One* 2:e455. 10.1371/journal.pone.0000455 17520014 PMC1866182

[B7] Ashwell K. W. S. McAllan B. M. Mai J. K. Paxinos G. (2008). Cortical cyto- and chemoarchitecture in three small Australian marsupial carnivores: *Sminthopsis macroura*, *Antechinus stuartii* and *Phascogale calura*. *Brain Behav. Evol.* 72 215–232. 10.1159/000165101 18946209

[B8] Balleza-Tapia H. Arroyo-García L. E. Isla A. G. Loera-Valencia R. Fisahn A. (2022). Functionally-distinct pyramidal cell subpopulations during gamma oscillations in mouse hippocampal area CA3. *Prog. Neurobiol.* 210:102213. 10.1016/j.pneurobio.2021.102213 34954329

[B9] Baron G. Stephan H. Frahm H. D. (1996a). *Comparative Neurobiology in Chiroptera: Brain Characeteristics in Taxonomic Units.* Basel: Birkhäuser Verlag.

[B10] Baron G. Stephan H. Frahm H. D. (1996b). *Comparative Neurobiology in Chiroptera: Brain Characteristics in Functional Systems, Ecoethological Adaptation, Adaptive Radiation and Evolution.* Basel: Birkhäuser Verlag.

[B11] Baron G. Stephan H. Frahm H. D. (1996c). *Comparative Neurobiology in Chiroptera: Macromorphology, Brain Structures, Tables and Atlases.* Basel: Birkhäuser Verlag.

[B12] Borzello M. Ramirez S. Treves A. Lee I. Scharfman H. Stark C. (2023). Assessments of dentate gyrus function: Discoveries and debates. *Nat. Rev. Neurosci.* 24 502–517. 10.1038/s41583-023-00710-z 37316588 PMC10529488

[B13] Buhl E. H. Dann J. F. (1991). Cytoarchitecture, neuronal composition, and entorhinal afferents of the flying fox hippocampus. *Hippocampus* 1 131–152. 10.1002/hipo.450010203 1727000

[B14] Cavegn N. van Dijk R. M. Menges D. Brettschneider H. Phalanndwa M. Chimimba C. T. (2013). Habitat-specific shaping of proliferation and neuronal differentiation in adult hippocampal neurogenesis of wild rodents. *Front. Neurosci.* 7:59. 10.3389/fnins.2013.00059 23616743 PMC3629335

[B15] Celio M. R. (1990). Calbindin D-28k and parvalbumin in the rat nervous system. *Neuroscience* 35 375–475. 10.1016/0306-4522(90)90091-h 2199841

[B16] Cembrowski M. S. Spruston N. (2019). Heterogeneity within classical cell types is the rule: Lessons from hippocampal pyramidal neurons. *Nat. Rev. Neurosci.* 20 193–204. 10.1038/s41583-019-0125-5 30778192

[B17] Chawla M. K. Guzowski J. F. Ramirez-Amaya V. Lipa P. Hoffman K. L. Marriott L. K. (2005). Sparse, environmentally selective expression of Arc RNA in the upper blade of the rodent fascia dentata by brief spatial experience. *Hippocampus* 15 579–586. 10.1002/hipo.20091 15920719

[B18] Cid E. Marquez-Galera A. Valero M. Gal B. Medeiros D. C. Navarron C. M. (2021). Sublayer- and cell-type-specific neurodegenerative transcriptional trajectories in hippocampal sclerosis. *Cell Rep.* 35:109229. 10.1016/j.celrep.2021.109229 34107264

[B19] Cole T. B. Wenzel H. J. Kafer K. E. Schwartzkroin P. A. Palmiter R. D. (1999). Elimination of zinc from synaptic vesicles in the intact mouse brain by disruption of the *ZnT3* gene. *Proc. Natl. Acad. Sci. U. S. A.* 96 1716–1721. 10.1073/pnas.96.4.1716 9990090 PMC15571

[B20] Culhane A. C. Thioulouse J. Perrière G. Higgins D. G. (2005). MADE4: An R package for multivariate analysis of gene expression data. *Bioinformatics* 21 2789–2790. 10.1093/bioinformatics/bti394 15797915

[B21] Danscher G. Zimmer J. (1978). An improved Timm sulphide silver method for light and electron microscopic localization of heavy metals in biological tissues. *Histochemistry* 55 27–40. 10.1007/BF00496691 76622

[B22] de Winter W. Oxnard C. E. (2001). Evolutionary radiations and convergences in the structural organization of mammalian brains. *Nature* 409 710–714. 10.1038/35055547 11217859

[B23] DeFelipe J. (1993). Neocortical neuronal diversity: Chemical heterogeneity revealed by colocalization studies of classic neurotransmitters, neuropeptides, calcium-binding proteins, and cell surface molecules. *Cereb. Cortex* 3 273–289. 10.1093/cercor/3.4.273 8104567

[B24] Deguchi Y. Donato F. Galimberti I. Cabuy E. Caroni P. (2011). Temporally matched subpopulations of selectively interconnected principal neurons in the hippocampus. *Nat. Neurosci.* 14 495–504. 10.1038/nn.2768 21358645

[B25] Denzinger A. Schnitzler H.-U. (2013). Bat guilds, a concept to classify the highly diverse foraging and echolocation behaviors of microchiropteran bats. *Front. Physiol.* 4:164. 10.3389/fphys.2013.00164 23840190 PMC3699716

[B26] Ding L. Balsamo G. Chen H. Blanco-Hernandez E. Zouridis I. S. Naumann R. (2022). Juxtacellular opto-tagging of hippocampal CA1 neurons in freely moving mice. *eLife* 11:e71720. 10.7554/eLife.71720 35080491 PMC8791633

[B27] Donato F. Chowdhury A. Lahr M. Caroni P. (2015). Early- and late-born parvalbumin basket cell subpopulations exhibiting distinct regulation and roles in learning. *Neuron* 85 770–786. 10.1016/j.neuron.2015.01.011 25695271

[B28] Donato F. Rompan S. B. Caroni P. (2013). Parvalbumin-expressing basket-cell network plasticity induced by experience regulates adult learning. *Nature* 504 272–276. 10.1038/nature12866 24336286

[B29] Dorph-Petersen K. A. Nyengaard J. R. Gundersen H. J. G. (2001). Tissue shrinkage and unbiased stereological estimation of particle number and size. *J. Microsc.* 204(Pt 3), 232–246. 10.1046/j.1365-2818.2001.00958.x 11903800

[B30] Eichenbaum H. (2017). On the integration of space, time, and memory. *Neuron* 95 1007–1018. 10.1016/j.neuron.2017.06.036 28858612 PMC5662113

[B31] Eilam-Altstädter R. Las L. Witter M. E. Ulanovsky N. (2021). *Stereotaxic Brain Atlas of the Egyptian Fruit Bat.* London: Academic Press.

[B32] Eliav T. Maimon S. R. Aljadeff J. Tsodyks M. Ginosar G. Las L. (2021). Multiscale representation of very large environments in the hippocampus of flying bats. *Science* 372:eabg4020. 10.1126/science.abg4020 34045327

[B33] Erwin S. R. Sun W. Copeland M. Lindo S. Spruston N. Cembrowski M. S. (2020). A sparse, spatially biased subtype of mature granule cell dominates recruitment in hippocampal-associated behaviors. *Cell Rep.* 31:107551. 10.1016/j.celrep.2020.107551 32348756

[B34] Fabene P. F. Correia L. Carvalho R. A. Cavalheiro E. A. Bentivoglio M. (2001). The spiny rat *Proechimys guyannensis* as a model of resistance to epilepsy: Chemical characterization of hippocampal cell populations and pilocarpine-induced changes. *Neuroscience* 104 979–1002. 10.1016/s0306-4522(01)00138-5 11457585

[B35] Filice F. Janickova L. Henzi T. Bilella A. Schwaller B. (2020). The parvalbumin hypothesis of autism spectrum disorder. *Front. Cell Neurosci.* 18:577525. 10.3389/fncel.2020.577525 33390904 PMC7775315

[B36] Filice F. Vörckel K. J. Sungur A. Ö Wöhr M. Schwaller B. (2016). Reduction in parvalbumin expression not loss of the parvalbumin-expressing GABA interneuron subpopulation in genetic parvalbumin and shank mouse models of autism. *Mol. Brain* 9:10. 10.1186/s13041-016-0192-8 26819149 PMC4729132

[B37] Fitting S. Booze R. M. Hasselrot U. Mactutus C. F. (2010). Dose-dependent long-term effects of Tat in the rat Hippocampal formation: A design-based stereological study. *Hippocampus* 20 469–480. 10.1002/Hipo.20648 19489004 PMC3841077

[B38] Fleming T. H. (1982). “Foraging strategies of plant-visiting bats,” in *Ecology of Bats*, ed. Kunz T. H. (Boston, MA: Springer), 287–325.

[B39] Forli A. Yartsev M. M. (2023). Hippocampal representation during collective spatial behaviour in bats. *Nature* 621 796–803. 10.1038/s41586-023-06478-7 37648869 PMC10533399

[B40] Freund T. F. Buzsáki G. (1996). Interneurons of the hippocampus. *Hippocampus* 6 347–470. 10.1002/(SICI)1098-106319966:4<347::AID-HIPO1<3.0.CO;2-I 8915675

[B41] Fujise N. Liu Y. Hori N. Kosaka T. (1998). Distribution of calretinin immunoreactivity in the mouse dentate gyrus: II. mossy cells, with special reference to their dorsoventral difference in calretinin immunoreactivity. *Neuroscience* 82 181–200. 10.1016/s0306-4522(97)00261-3 9483514

[B42] Gall C. (1990). “Comparative anatomy of the hippocampus. With special reference to differences in the distributions of neuroactive peptides,” in *Comparative Structure and Evolution of Cerebral Cortex, Part II*, eds Jones E. G. Peters A. (New York, NY: Plenum Press), 167–213.

[B43] Gallitano A. L. Satvat E. Gil M. Marrone D. F. (2016). Distinct dendritic morphology across the blades of the rodent dentate gyrus. *Synapse* 70 277–282. 10.1002/syn.21900 26926290 PMC4879091

[B44] García-Cabezas M. Á John Y. J. Barbas H. Zikopoulos B. (2016). Distinction of neurons, glia and endothelial cells in the cerebral cortex: An algorithm based on cytological features. *Front. Neuroanat.* 10:107. 10.3389/fnana.2016.00107 27847469 PMC5088408

[B45] Gatome C. W. Mwangi D. K. Lipp H. P. Amrein I. (2010). Hippocampal neurogenesis and cortical cellular plasticity in Wahlberg’s Epauletted Fruit Bat: A qualitative and quantitative study. *Brain Behav. Evol.* 76 116–127. 10.1159/000320210 20948188

[B46] Geiller T. Fattahi M. Choi J.-S. Royer S. (2017). Place cells are more strongly tied to landmarks in deep than in superficial CA1. *Nat. Commun.* 8:14531. 10.1038/ncomms14531 28218283 PMC5321734

[B47] Geva-Sagiv M. Romani S. Las L. Ulanovsky N. (2016). Hippocampal global remapping for different sensory modalities in flying bats. *Nat. Neurosci.* 19 952–958. 10.1038/nn.4310 27239936

[B48] Graf R. F. Fischer C. (2021). *Atlas der Säugetiere. Schweiz und Lichtenstein. Schweizerische Gesellschaft für Wildtierbiologie SGW.* Bern: Haupt Verlag.

[B49] Gulyás A. I. Miettinen R. Jacobowitz D. M. Freund T. F. (1992). Calretinin is present in non-pyramidal cells of the rat hippocampus - I. a new type of neuron specifically associated with the mossy fibre system. *Neuroscience* 48 1–27. 10.1016/0306-4522(92)90334-X 1584417

[B50] Gundersen H. J. G. Jensen E. B. V. Kieu K. Nielsen J. (1999). The efficiency of systematic sampling in stereology – reconsidered. *J. Microsc.* 193 199–211. 10.1046/j.1365-2818.1999.00457.x 10348656

[B51] Gurden H. Schiffmann S. N. Lemaire M. Böhme G. A. Parmentier M. Schurmans S. (1998). Calretinin expression as a critical component in the control of dentate gyrus long-term potentiation induction in mice. *Eur J Neurosci* 10 3029–3033. 10.1111/j.1460-9568.1998.00373.x 9758174

[B52] Harvey R. E. Robinson H. L. Liu C. Oliva A. Fernandez-Ruiz A. (2023). Hippocampo-cortical circuits for selective memory encoding, routing, and replay. *Neuron* 111 2076–2090.e9. 10.1016/j.neuron.2023.04.015 37196658 PMC11146684

[B53] Hof P. R. Rosenthal R. E. Fiskum G. (1996). Distribution of neurofilament protein and calcium-binding proteins parvalbumin, calbindin, and calretinin in the canine hippocampus. *J. Chem. Neuroanat.* 11 1–12. 10.1016/0891-0618(96)00117-2 8841885

[B54] Holm I. E. Geneser F. A. Zimmer J. (1993). Cholecystokinin-, enkephalin-, and substance P-like immunoreactivity in the dentate area, hippocampus, and subiculum of the domestic pig. *J. Comp. Neurol.* 331 310–325. 10.1002/cne.903310303 7685777

[B55] Huszár R. Zhang Y. Blockus H. Buzsáki G. (2022). Preconfigured dynamics in the hippocampus are guided by embryonic birthdate and rate of neurogenesis. *Nat. Neurosci.* 25 1201–1212. 10.1038/s41593-022-01138-x 35995878 PMC10807234

[B56] Hutcheon J. M. Kirsch J. A. Garland T. Jr. (2002). A comparative analysis of brain size in relation to foraging ecology and phylogeny in the Chiroptera. *Brain Behav. Evol.* 60 165–180. 10.1159/000065938 12417821

[B57] Imam A. Bhagwandin A. Ajao M. S. Ihunwo A. O. Manger P. R. (2019). The brain of the tree pangolin (*Manis tricuspis*). IV. The hippocampal formation. *J. Comp. Neurol.* 527 2393–2412. 10.1002/cne.24620 30592043

[B58] Imura T. Kobayashi Y. Suzutani K. Ichikawa-Tomikawa N. Chiba H. (2019). Differential expression of a stress-regulated gene Nr4a2 characterizes early- and late-born hippocampal granule cells. *Hippocampus* 29 539–549. 10.1002/hipo.23045 30365199

[B59] Iñiguez C. Gayoso M. J. Carreres J. (1985). A versatile and simple method for staining nervous tissue using Giemsa dye. *J. Neurosci. Meth.* 13 77–86. 10.1016/0165-0270(85)90045-7 3887046

[B60] Ishihara Y. Fukuda T. (2016). Immunohistochemical investigation of the internal structure of the mouse subiculum. *Neuroscience* 337 242–266. 10.1016/j.neuroscience.2016.09.027 27664459

[B61] Jacobs M. S. McFarland W. L. Morgane P. J. (1979). The anatomy of the brain of the Bottlenose dophin (*Tursiops truncatus*). Rhinic lobe (rhinencephalon): The archicortex. *Brain Res. Bull* 4 (Suppl. 1), 1–108. 10.1016/0361-9230(79)90299-5 551842

[B62] Jacobsen B. Kleven H. Gatome W. Las L. Ulanovsky N. Witter M. P. (2023). Organization of projections from the entorhinal cortex to the hippocampal formation of the Egyptian fruit bat *Rousettus aegyptiacus*. *Hippocampus* 33 889–905. 10.1002/hipo.23517 36869437

[B63] Jinno S. Kosaka T. (2006). Cellular architecture of the mouse hippocampus: A quantitative aspect of chemically defined GABAergic neurons with stereology. *Neurosci. Res.* 56 229–245. 10.1016/j.neures.2006.07.007 16930755

[B64] Johnston D. Amaral D. G. (1998). “Hippocampus,” in *The Synaptic Organization of the Brain*, 4th Edn, ed. Shepherd G. M. (New York, NY: Oxford University Press).

[B65] Jouvenceau A. Poitier P. Battini R. Ferrari S. Dutar P. Billard J. M. (1999). Glutamatergic synaptic responses and long-term potentiation are impaired in the CA1 hippocampal area of calbindin D_28k_-deficient mice. *Synapse* 33 172–180. 10.1002/(SICI)1098-2396(19990901)33:3<172::AID-SYN2<3.0.CO;2-S 10420165

[B66] Jouvenceau A. Potier B. Poindessous-Jazat F. Dutar P. Slama A. Epelbaum J. (2002). Decrease in calbindin content significantly alters LTP but not NMDA receptor and calcium channel properties. *Neuropharmacology* 42 444–458. 10.1016/s0028-3908(01)00202-7 11955516

[B67] Karunakaran S. Chowdhury A. Donato F. Quairiaux C. Michel C. M. Caroni P. (2016). PV plasticity sustained through D1/5 dopamine signaling required for long-term memory consolidation. *Nat. Neurosci.* 19 454–464. 10.1038/nn.4231 26807952

[B68] Krook-Magnuson E. Varga C. Lee S.-H. Soltesz I. (2012). New dimensions of interneuronal specialization unmasked by principal cell heterogeneity. *Trends Neurosci.* 35 175–184. 10.1016/j.tins.2011.10.005 22119146 PMC3294038

[B69] Le Roux N. Cabezas C. Böhm U. L. Poncer J. C. (2013). Input-specific learning rules at excitatory synapses onto hippocampal parvalbumin-expressing interneurons. *J. Physiol.* 591 1809–1822. 10.1113/jphysiol.2012.245852 23339172 PMC3624852

[B70] Lever C. Burton S. Jeewajee A. Keefe J. Burgess N. (2009). Boundary vector cells in the subiculum of the hippocampal formation. *J. Neurosci.* 29:9771. 10.1523/JNEUROSCI.1319-09.2009 19657030 PMC2736390

[B71] Li J. T. Xie X. M. Yu J. Y. Sun Y. X. Liao X. M. Wang X. X. (2017). Suppressed calbindin levels in hippocampal excitatory neurons mediate stress-induced memory loss. *Cell Rep.* 21 891–900. 10.1016/j.celrep.2017.10.006 29069596

[B72] Liu Y. Fujise N. Kosaka T. (1996). Distribution of calretinin in the mouse dentate gyrus I. general description. *Exp. Brain Res.* 108 389–403. 10.1007/BF00227262 8801119

[B73] Lorente de Nó R. (1934). Studies on the structure of the cerebral cortex II. continuation of the study of the Ammonic system. *J. Psychol. Neurol.* 46 113–177.

[B74] Lucas E. K. Markwardt S. J. Gupta S. Meador-Woodruff J. H. Lin J. D. Overstreet-Wadiche L. (2010). Parvalbumin deficiency and GABAergic dysfunction in mice lacking PGC-1alpha. *J. Neurosci.* 30 7227–7235. 10.1523/JNEUROSCI.0698-10.2010 20505089 PMC2888101

[B75] Maliković J. Amrein I. Vinciguerra L. Lalošević D. Wolfer D. P. Slomianka L. (2023). Cell numbers in the reflected blade of CA3 and their relation to other hippocampal principal cell populations across seven species. *Front. Neuroanat.* 16:1070035. 10.3389/fnana.2022.1070035 36686574 PMC9846821

[B76] Maliković J. Amrein I. Vinciguerra L. Wolfer D. P. Slomianka L. (2024). NECAB1-3, parvalbumin, calbindin, and calretinin in the hippocampus of the European mole. *Front Neuroanat* 18:1452722. 10.3389/fnana.2024.1452722 39296922 PMC11408328

[B77] Marissal T. Bonifazi P. Picardo M. A. Nardou R. Petit L. F. Baude A. (2012). Pioneer glutamatergic cells develop into a morpho-functionally distinct population in the juvenile CA3 hippocampus. *Nat. Commun.* 3:1316. 10.1038/ncomms2318 23271650 PMC3535425

[B78] Matsumoto N. Kitanishi T. Mizuseki K. (2019). The subiculum: Unique hippocampal hub and more. *Neurosci. Res.* 143 1–12. 10.1016/j.neures.2018.08.002 30121285

[B79] Medalla M. Mo B. Nasar R. Zhou Y. Park J. Luebke J. I. (2023). Comparative features of calretinin, calbindin, and parvalbumin expressing interneurons in mouse and monkey primary visual and frontal cortices. *J. Comp. Neurol.* 531 1934–1962. 10.1002/cne.25514 37357562 PMC10749991

[B80] Mishra P. Narayanan R. (2020). Heterogeneities in intrinsic excitability and frequency-dependent response properties of granule cells across the blades of the rat dentate gyrus. *J. Neurophysiol.* 123 755–772. 10.1152/jn.00443.2019 31913748 PMC7052640

[B81] Molinari S. Battini R. Ferrari S. Pozzi L. Killcross A. S. Robbins T. W. (1996). Deficits in memory and hippocampal long-term potentiation in mice with reduced calbindin D_28K_ expression. *Proc. Natl. Acad. Sci. U. S. A.* 93 8028–8033. 10.1073/pnas.93.15.8028 8755597 PMC38869

[B82] Murakawa R. Kosaka T. (1999). Diversity of the calretinin immunoreactivity in the dentate gyrus of gerbils, hamsters, guinea pigs and laboratory shrews. *J. Comp. Neurol.* 411 413–430. 10.1002/(SICI)1096-9861(19990830)411:3<413::AID-CNE5<3.0.CO;2-Q 10413776

[B83] Murray A. J. Sauer J. F. Riedel G. McClure C. Ansel L. Cheyne L. (2011). Parvalbumin-positive CA1 interneurons are required for spatial working but not for reference memory. *Nat. Neurosci.* 14:297. 10.1038/nn.2751 21278730 PMC3064406

[B84] Norberg U. M. Rayner J. M. V. (1987). Ecological morphology of flight in bats (Mammalia; Chiroptera): Wing adaptations, flight performance, foraging strategy and echolocation. *Philos. Trans. R. Soc. Lond. B Biol. Sci.* 316 335–427. 10.1098/rstb.1987.0030

[B85] Paradis E. Schliep K. (2019). ape 5.0: An environment for modern phylogenetics and evolutionary analyses in R. *Bioinformatics* 35 526–528. 10.1093/bioinformatics/bty633 30016406

[B86] Pasti L. Carmignoto G. Pozzan T. Battini R. Ferrari S. Lally G. (1999). Cellular calcium handling in brain slices from calbindin D_28k_-deficient mice. *Neuroreport* 10 2367–2372. 10.1097/00001756-199908020-00027 10439465

[B87] Pillay S. Bhagwandin A. Bertelsen M. F. Patzke N. Engler G. Engel A. K. (2021). The hippocampal formation of two carnivore species: The feliform banded mongoose and the caniform domestic ferret. *J. Comp. Neurol.* 529 8–27. 10.1002/cne.25047 33016331

[B88] Poulter S. Lee S. A. Dachtler J. Wills T. J. Lever C. (2021). Vector trace cells in the subiculum of the hippocampal formation. *Nat. Neurosci.* 24 266–275. 10.1038/s41593-020-00761-w 33349710 PMC7116739

[B89] Rami A. Brehier A. Thomasset M. Rabie A. (1987). The comparative immunocytochemical distribution of 28kDa cholecalcin (CaBP) in the hippocampus of rat, guinea pig and hedgehog. *Brain Res.* 422 149–153. 10.1016/0006-8993(87)90549-x 3676777

[B90] Ramírez-Amaya V. Vazdarjanova A. Mikhael D. Rosi S. Worley P. F. Barnes C. A. (2005). Spatial exploration-induced *Arc* mRNA and protein expression: Evidence for selective, network-specific reactivation. *J. Neurosci.* 25 1761–1768. 10.1523/jneurosci.4342-04.2005 15716412 PMC6725922

[B91] Ratcliffe J. M. (2009). Neuroecology and diet selection in phyllostomid bats. *Behav. Process.* 80 247–251. 10.1016/j.beproc.2008.12.010 20522315

[B92] Ratcliffe J. M. Fenton M. B. Shettleworth S. J. (2006). Behavioral flexibility positively correlated with relative brain volume in predatory bats. *Brain Behav Evol* 67 165–176. 10.1159/000090980 16415571

[B93] Ray S. Yona I. Elami N. Palgi S. Latimer K. W. Jacobsen B. (2025). Hippocampal coding of identity, sex, hierarchy, and affiliation in a social group of wild fruit bats. *Science* 387 eadk9385. 10.1126/science.adk9385 39883756

[B94] Rebola N. Carta M. Mulle C. (2017). Operation and plasticity of hippocampal CA3 circuits: implications for memory encoding. *Nat. Rev. Neurosci.* 18 208–220. 10.1038/nrn.2017.10 28251990

[B95] Rekart J. L. Routtenberg A. (2010). Overexpression of GAP-43 reveals unexpected properties of hippocampal mossy fibers. *Hippocampus* 20 46–57. 10.1002/hipo.20668 19650124 PMC2874863

[B96] Revell L. J. (2024). phytools 2.0: An updated R ecosystem for phylogenetic comparative methods (and other things). *Peer J* 12 e16505. 10.7717/peerj.16505 38192598 PMC10773453

[B97] Rosene D. L. van Hoesen G. W. (1987). “The hippocampal formation of the primate brain. A review of some comparative aspects of cytoarchitecture and connections,” in *Cerebral cortex*, eds Jones E. G. Peters A. (New York, NY: Plenum Publishing Corporation), 345–456.

[B98] Safi K. Dechmann D. K. (2005). Adaptation of brain regions to habitat complexity: A comparative analysis in bats (Chiroptera). *Proc. R. Soc. B.* 272 179–186. 10.1098/rspb.2004.2924 15695209 PMC1634959

[B99] Sarel A. Finkelstein A. Las L. Ulanovsky N. (2017). Vectorial representation of spatial goals in the hippocampus of bats. *Science* 355 176–180. 10.1126/science.aak9589 28082589

[B100] Satvat E. Schmidt B. Argraves M. Marrone D. F. Markus E. J. (2011). Changes in task demands alter the pattern of *zif268* expression in the dentate gyrus. *J. Neurosci.* 31:7163. 10.1523/JNEUROSCI.0094-11.2011 21562279 PMC6703202

[B101] Save L. Baude A. Cossart R. (2019). Temporal embryonic origin critically determines cellular physiology in the dentate gyrus. *Cereb. Cortex* 29 2639–2652. 10.1093/cercor/bhy132 29878074

[B102] Schmidt B. Marrone D. F. Markus E. J. (2012). Disambiguating the similar: The dentate gyrus and pattern separation. *Behav. Brain Res.* 226 56–65. 10.1016/j.bbr.2011.08.039 21907247

[B103] Schwaller B. (2020). Cytosolic Ca^2+^ buffers are inherently Ca^2+^ signal modulators. *Cold Spring Harb. Perspect. Biol.* 12:a035543. 10.1101/cshperspect.a035543 31308146 PMC6942125

[B104] Seress L. Ábrahám H. Czéh B. Fuchs E. Léránth C. (2008). Calretinin expression in hilar mossy cells of the hippocampal dentate gyrus of nonhuman primates and humans. *Hippocampus* 18 425–434. 10.1002/hipo.20403 18189312

[B105] Seress L. Gulyas A. I. Freund T. F. (1991). Parvalbumin- and calbindin D_28k_-immunoreactive neurons in the hippocampal formation of the macaque monkey. *J. Comp. Neurol.* 313 162–177. 10.1002/cne.903130112 1761752

[B106] Shah S. Lubeck E. Zhou W. Cai L. (2016). In situ transcription profiling of single cells reveals spatial organization of cells in the mouse hippocampus. *Neuron* 92 342–357. 10.1016/j.neuron.2016.10.001 27764670 PMC5087994

[B107] Sharif F. Tayebi B. Buzsáki G. Royer S. Fernandez-Ruiz A. (2021). Subcircuits of deep and superficial CA1 place cells support efficient spatial coding across heterogeneous environments. *Neuron* 109 363–376.e6. 10.1016/j.neuron.2020.10.034 33217328 PMC7856084

[B108] Slomianka L. (2021). Basic quantitative morphological methods applied to the central nervous system. *J. Comp. Neurol.* 529 694–756. 10.1002/cne.24976 32639600 PMC7818269

[B109] Slomianka L. Amrein I. Knuesel I. Sorensen J. C. Wolfer D. P. (2011). Hippocampal pyramidal cells: The reemergence of cortical lamination. *Brain Struct. Funct.* 216 301–317. 10.1007/s00429-011-0322-0 21597968 PMC3197924

[B110] Slomianka L. Drenth T. Cavegn N. Menges D. Lazic S. E. Phalanndwa M. (2013). The hippocampus of the eastern rock sengi: Cytoarchitecture, markers of neuronal function, principal cell numbers and adult neurogenesis. *Front. Neuroanat.* 7:34. 10.3389/fnana.2013.00034 24194702 PMC3810719

[B111] Sloviter R. S. (1989). Calcium-binding protein (calbindin-D_28k_) immunocytochemistry: Localization in the rat hippocampus with specific reference to the selective vulnerability of hippocampal neurons to seizure activity. *J. Comp. Neurol.* 280 183–196. 10.1002/cne.902800203 2925892

[B112] Soltesz I. Losonczy A. (2018). CA1 pyramidal cell diversity enabling parallel information processing in the hippocampus. *Nat. Neurosci.* 21 484–493. 10.1038/s41593-018-0118-0 29593317 PMC5909691

[B113] Stark E. Roux L. Eichler R. Senzai Y. Royer S. Buzsáki G. (2014). Pyramidal cell-interneuron interactions underlie hippocampal ripple oscillations. *Neuron* 83 467–480. 10.1016/j.neuron.2014.06.023 25033186 PMC4393648

[B114] Stephan H. (1975). *Allocortex.* Berlin: Springer Verlag.

[B115] Stephan H. Frahm H. D. Baron G. (1987). Brains of Vespertilionids III. Comparative cytoarchitectonics of the hippocampus. *Z. Zool. Syst. Evolut. Forsch.* 25 205–211. 10.1111/j.1439-0469.1987.tb00604.x

[B116] Strange B. A. Witter M. P. Lein E. S. Moser E. I. (2014). Functional organization of the hippocampal longitudinal axis. *Nat Rev Neurosci* 15 655–669. 10.1038/nrn3785 25234264

[B117] Strauch C. Böge J. Shchyglo O. Dubovyk V. Manahan-Vaughan D. (2025). The suprapyramidal and infrapyramidal blades of the dentate gyrus exhibit different GluN subunit content and dissimilar frequency-dependent synaptic plasticity in Vivo. *Hippocampus* 35:e70002. 10.1002/hipo.70002 39994965 PMC11850964

[B118] Todkar K. Scotti A. L. Schwaller B. (2012). Absence of the calcium-binding protein calretinin, not of calbindin D-28k, causes a permanent impairment of murine adult hippocampal neurogenesis. *Front. Mol. Neurosci.* 5:56. 10.3389/fnmol.2012.00056 22536174 PMC3332231

[B119] Ulanovsky N. Moss C. F. (2007). Hippocampal cellular and network activity in freely moving echolocating bats. *Nat. Neurosci.* 10 224–233. 10.1038/nn1829 17220886

[B120] Valero M. de la Prida L. M. (2018). The hippocampus in depth: A sublayer-specific perspective of entorhinal–hippocampal function. *Curr. Opin. Neurobiol.* 52 107–114. 10.1016/j.conb.2018.04.013 29729527

[B121] van Dijk R. M. Huang S.-H. Slomianka L. Amrein I. (2016). Taxonomic separation of hippocampal networks: Principal cell populations and adult neurogenesis. *Front. Neuroanat.* 10:22. 10.3389/fnana.2016.00022 27013984 PMC4783399

[B122] van Tussenbroek I. A. Knörnschild M. Nagy M. ten Cate C. J. Vernes S. C. (2023). Morphological diversity in the brains of 12 Neotropical bat species. *Acta Chiropterol.* 25 323–338. 10.3161/15081109ACC2023.25.2.011

[B123] Vreugdenhil M. Jefferys J. G. R. Celio M. R. Schwaller B. (2003). Parvalbumin-deficiency facilitates repetitive IPSCs and gamma oscillations in the hippocampus. *J. Neurophysiol.* 89 1414–1422. 10.1152/jn.00576.2002 12626620

[B124] West M. J. Slomianka L. Gundersen H. J. (1991). Unbiased stereological estimation of the total number of neurons in the subdivisions of the rat hippocampus using the optical fractionator. *Anat. Rec.* 231 482–497. 10.1002/ar.1092310411 1793176

[B125] Wickham H. (2016). *ggplot2: Elegant graphics for data analysis.* New York, NY: Springer Verlag.

[B126] Yartsev M. M. Ulanovsky N. (2013). Representation of three-dimensional space in the hippocampus of flying bats. *Science* 340 367–372. 10.1126/science.1235338 23599496

[B127] Yu C. Moss C. F. (2022). Natural acoustic stimuli evoke selective responses in the hippocampus of passive listening bats. *Hippocampus* 32 298–309. 10.1002/hipo.23407 35085416 PMC9306857

[B128] Yu G. Lam T. T.-Y. Zhu H. Guan Y. (2018). Two methods for mapping and visualizing associated data on phylogeny using ggtree. *Mol. Biol. Evol.* 35 3041–3043. 10.1093/molbev/msy194 30351396 PMC6278858

