# Supplementary material for: Hippocampal structure, patterns of the calcium-binding proteins and neuron numbers in small echolocating bats
Source: Front Neuroanat. 2025 Aug 13;19:1641787. doi: 10.3389/fnana.2025.1641787 (PMC12380690; doi:10.3389/fnana.2025.1641787)
Supplement: Supplementary file 1 [file Data_Sheet_1.pdf]

Supplementary Material

Hippocampal structure, patterns of the calcium-binding proteins and neuron numbers in small echolocating bats

Jovana Maliković[1], Katja Schönbächler[2], Ana Luiza F. Destro[3], David P. Wolfer[1,4], Irmgard Amrein[1,4*]

[1]Division of Functional Neuroanatomy, Institute of Anatomy, University of Zürich, Zürich, Switzerland

[2] Bat Conservation Switzerland, Zürich, Switzerland

[3]Department of Animal Biology, Federal University of Viçosa, Minas Gerais, Brazil

[4]Institute of Human Movement Sciences and Sport, D-HEST, ETH Zürich, Switzerland

Supplementary Figure 1

Morphology of CA3 pyramidal neurons in *Phyllostomus discolor*

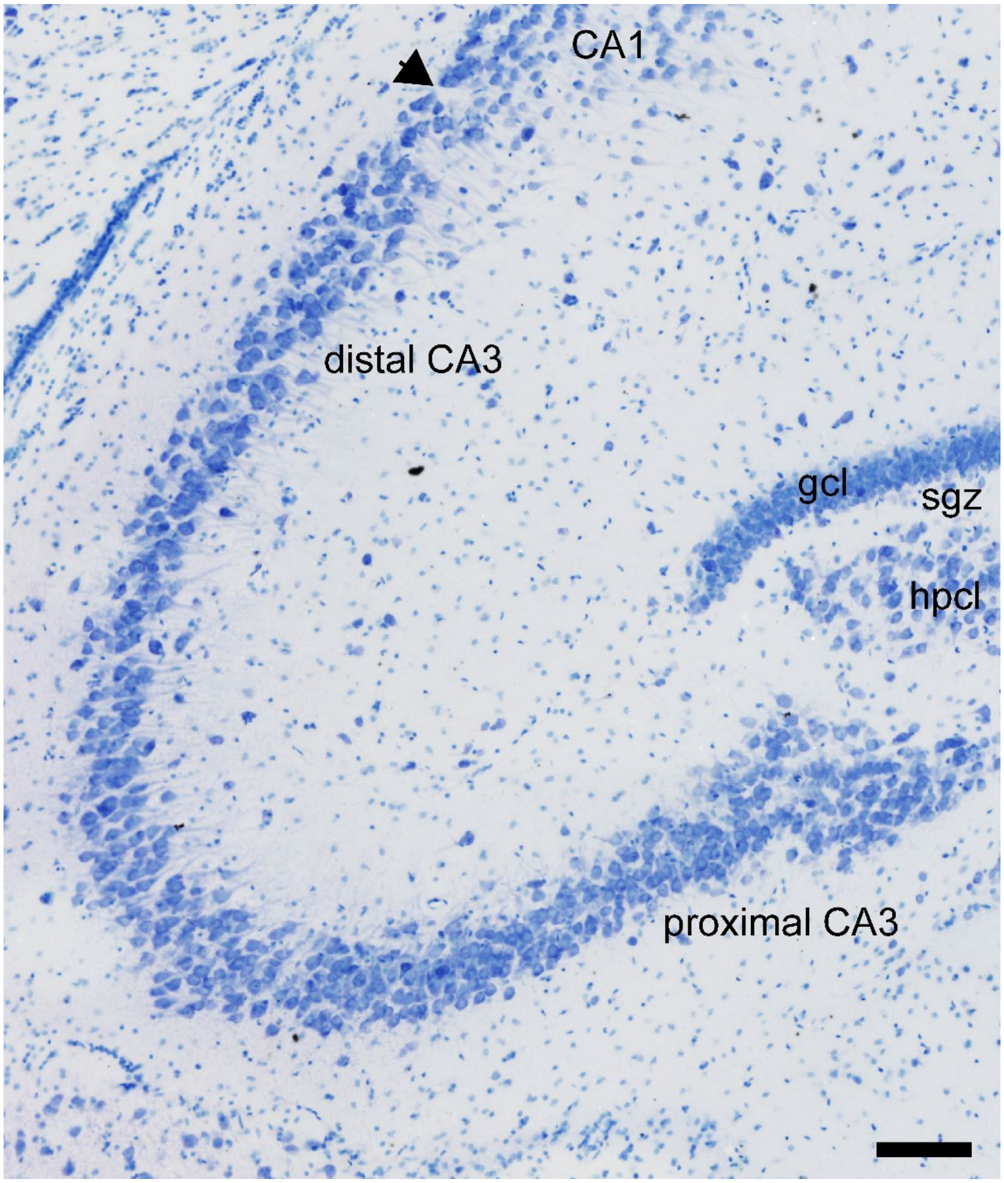

A Nissl stained horizontal section of the hippocampus at septal level in *Phyllostomus discolor* reveals two morphologically distinct CA3 pyramidal neurons. In proximal CA3, pyramidal neurons appear smaller and with shorter apical dendrites than in distal CA3. Scale bar: 100µm. Abbreviations: gcl: granule cell layer; sgz: subgranular zone; hpcl: hilar polymorphic cell layer. Black arrow indicates boundary between CA3 and CA1.

Supplementary Figure 2

Log-linear relations of hippocampal neuron numbers with brain weight

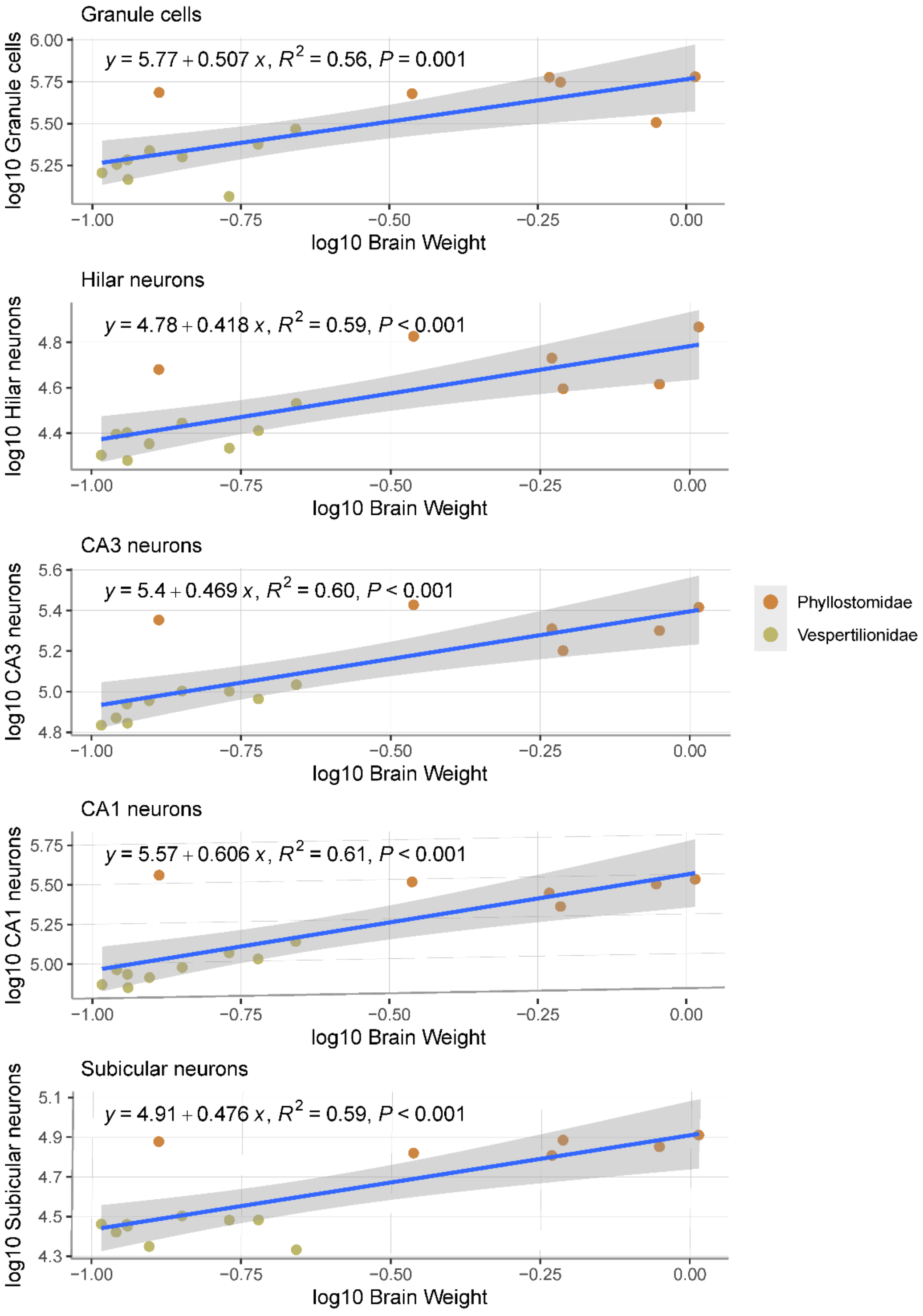

Supplementary Figure 3

Additional IHC illustrations

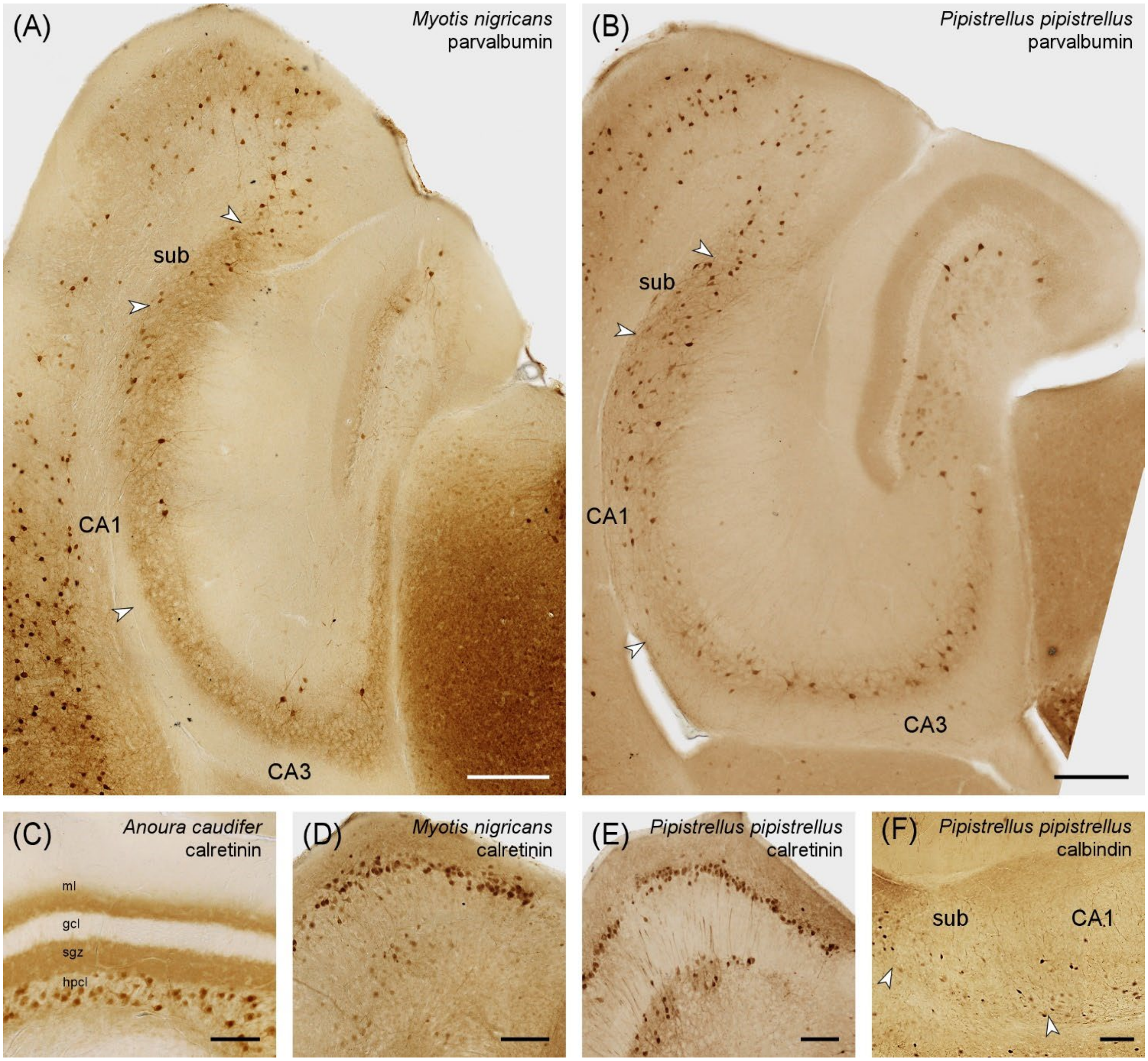

(**A**) Parvalbumin stainings in *Myotis nigricans* and (**B**) *Pipistrellus pipistrellus* emphasize the consistent expression pattern of this protein in bats. (**C**) CR+ expression in the dentate gyrus of *Anoura caudifer* was lighter than in *Phyllostomus discolor* and a staining of the middle molecular layer is missing. (**D**) Strong CR+ staining of neurons in MEC layer ll in *Myotis nigricans* and (**E**) *Pipistrellus pipistrellus* emphasize the common feature of this pattern in vespertilionids. (**F**) Calb+ staining in the subiculum of *Pipistrellus pipistrellus* is weaker than in *Vespertilio murinus*. Scalebar: A, B: 200µm, C-F: 100µm. Abbreviations: sub: subiculum; ml: molecular layer of the dentate gyrus;

gcl: granule cell layer; sgz: subgranular zone; hpcl: hilar polymorphic cell layer. White arrows mark neuron population boundaries between CA3, CA1 and subiculum.

Supplementary Table 1

Summary table of sampling parameters applied to the species using the optical fractionator method. Disector height of 10µm, with a guard zone of 2µm, was used for all subregions and species.

**Vespertilionidae**	***Myotis daubentonii***	***Myotis mystacinus***	***Myotis nigricans***	***Pipistrellus kuhlii***	***Pipistrellus nathusii***	***Pipistrellus pipistrellus***	***Pipistrellus pygmaeus***	***Plecotus auritus***	***Vespertilio murinus***
Section sampling fraction	1/6th and 1/8th	1/6th	1/4th	1/4th	1/4th and 1/9th	1/4th and 1/6th	1/4th	1/6th	1/4th
Range of section numbers	17-24	19	23	27-32	16-29	18-31	28	25	26
Granule cells									
Counting frame, µm	15x15	15x15	15x15	10x10	10x10	10x10	15x15	15x15	15x15
Step size, µm	150	180	150	120	120	120	180	180	150
Counts, mean (SD)	163 (36)	177 (24)	151 (62)	205 (47)	165 (48)	153 (27)	144	221	159
Hilar neurons									
Counting frame, µm	50x50	50x50	50x50	50x50	50x50	50x50	50x50	50x50	50x50
Step size, µm	180	180	150	120	120	120	180	180	150
Counts, mean (SD)	155 (16)	184 (7)	183 (50)	628 (206)	558 (150)	453 (88)	184	266	286
CA3 pyramidal neurons									
Counting frame, µm	25x25	25x25	25x25	25x25	25x25	25x25	25x25	25x25	25x25
Step size, µm	180	180	180	160	160	160	180	180	180
Counts, mean (SD)	148 (20)	182 (7)	145 (19)	311 (74)	288 (82)	227 (36)	145	217	231
CA1 pyramidal neurons									
Counting frame, µm	25x25	25x25	25x25	25x25	25x25	25x25	25x25	25x25	25x25
Step size, µm	180	180	180	160	160	160	180	180	180
Counts, mean (SD)	166 (44)	169 (13)	158 (7)	293 (78)	273 (83)	239 (14)	162	259	249
Subicular neurons									
Counting frame, µm	25x25	25x25	25x25	25x25	25x25	25x25	25x25	25x25	25x25
Step size, µm	120	120	120	120	120	120	120	120	120
Counts, mean (SD)	185 (133)	103 (2)	135 (23)	180 (50)	168 (66)	169 (26)	117	96	164

**Phyllostomidae**	***Anoura caudifer***	***Carollia perspicillata***	***Desmodus rotundus***	***Diphylla ecaudata***	***Phyllostomus discolor***	***Sturnira lilium***
Section sampling fraction	1/10th	1/12th	1/9th	1/9th	1/9th and 1/12th	1/10th
Range of section numbers	19-22	17-19	20-27	23-27	21-26	16-24
Granule cells						
Counting frame, µm	15x15	15x15	15x15	15x15	10x10	15x15
Step size, µm	180	140	120	180	120	180
Counts, mean (SD)	200 (20)	303 (79)	243 (114)	491 (236)	209 (32)	178 (41)
Hilar neurons						
Counting frame, µm	50x50	60x60	50x50	50x50	60x60	50x50
Step size, µm	180	140	180	180	120	180
Counts, mean (SD)	204 (12)	403 (82)	180 (19)	176 (32)	733 (242)	253 (5)
CA3 pyramidal neurons						
Counting frame, µm	25x25	30x30	25x25	25x25	25x25	25x25
Step size, µm	180	150	180	180	160	180
Counts, mean (SD)	222 (43)	427 (227)	220 (53)	188 (30)	318 (42)	260 (19)
CA1 pyramidal neurons						
Counting frame, µm	25x25	30x30	25x25	25x25	25x25	25x25
Step size, µm	180	200	180	180	160	180
Counts, mean (SD)	398 (31)	325 (172)	352 (23)	275 (46)	431 (61)	330 (47)
Subicular neurons						
Counting frame, µm	25x25	30x30	25x25	25x25	25x25	25x25
Step size, µm	120	100	120	120	120	120
Counts, mean (SD)	188 (35)	393 (317)	172 (12)	205 (20)	179 (18)	147 (34)
